# Emergy-based evaluation of production efficiency and sustainability of diversified multi-cropping systems in the Yangtze River Basin

**DOI:** 10.3389/fpls.2024.1454130

**Published:** 2024-11-08

**Authors:** Xinhui Lei, Bing Liang, Liang Feng, Xuyang Zhao, Tian Pu, Changbing Yu, Shubin Wang, Yafeng Wei, Shumei Ma, Xiaochun Wang, Wenyu Yang

**Affiliations:** ^1^ College of Agronomy, Sichuan Agricultural University, Chengdu, China; ^2^ Sichuan Engineering Research Center for Crop Strip Intercropping System, Key Laboratory of Crop Ecophysiology and Farming System in Southwest China (Ministry of Agriculture), Chengdu, China; ^3^ Institute of Oil Crops, Chinese Academy of Agricultural Sciences, Wuhan, China; ^4^ College of Agriculture, Jiangxi Agricultural University, Nanchang, China; ^5^ Economic Crop Research Office, Jiangsu Yanjiang Institute of Agricultural Sciences, Nantong, China; ^6^ Institute of Crops Research, Hunan Academy of Agricultural Sciences, Changsha, China

**Keywords:** emergy analysis, environmental sustainability, multi-cropping systems, strip compound planting, Yangtze River Basin

## Abstract

Excessive agricultural investment brought about by increased multiple-cropping index may compromise environmental sustainability. There are few studies on the sustainability of diversified multi-cropping systems in the Yangtze River Basin (YRB). Therefore, this study selected five representative locations in the YRB. According to the local climate characteristics and food demand, diversified multi-cropping systems were designed, and the main local winter crops were selected as the previous crops of the corn–soybean strip compound cropping system, with the local traditional double-cropping model as the control (CK). The emergy evaluation method was introduced to quantitatively compare the efficiency and sustainability of diversified multi-cropping systems in the YRB. The results showed that by incorporating soybean by intercropping with corn, compared with the CK, the total energy input, annual energy output, and annual economic output increased by 15.80%, 9.78%, and 33.12% on average, respectively. The unit emergy value (UEV) and unit non-renewable value (UNV) increased by 6.03% and 5.98%, respectively; the emergy yield ratio (EYR) and environmental loading ratio (ELR) decreased by 0.91% and 0.44%, respectively; the emergy sustainability index (ESI) was the same. In the third mature crop selection, compared with that of corn, the ELR of soybean decreased by 14.32%, and the ESI increased by 18.55%. In addition, the choice of winter crops plays a vital role in the system’s efficiency and sustainability. Compared with those of other winter crops, the annual economic outputs of potato (upper reaches of the YRB), potato or forage rape (middle reaches of the YRB), and wheat (lower reaches of the YRB) increased by 51.02%, 32.27%, and 0.94%, respectively; their ESI increased by 71.21%, 47.72%, and 12.07%, respectively. Potato–corn/soybean or potato/corn/soybean (upper reaches of the YRB), forage rape–corn/soybean or potato/corn/soybean (middle reaches of the YRB), and wheat–corn/soybean (lower reaches of the YRB) were chosen to facilitate the coexistence of high economic benefits and environmental sustainability. Additionally, promoting mechanization and reducing labor input were essential to improve the efficiency and sustainability of multi-cropping systems. This study would provide a scientific basis and theoretical support for the development of efficient and sustainable multiple-cropping systems in the dryland of the YRB.

## 1 Introduction

Facing the challenges of global population growth, food security, and environmental pressure, it is imperative to develop sustainable agriculture (Dai et al., 2024; Dong and Liu, 2023). As the largest developing country and agricultural country in the world, China is under significant pressure (Gao et al., 2023; Guo et al., 2021; He et al., 2019; Yang et al., 2019). However, the potential of developing new cultivated land is severely limited, so it is necessary to increase the grain output per unit area of existing farmland to meet the growing demand for grain. Enhancing the multiple-cropping index and optimizing resource use will play a key role in achieving this objective (Long et al., 2022; Yu and Deng, 2022). The Yangtze River Basin is rich in light and heat resources, with an average annual temperature of 16°C–18°C and rainfall of approximately 1,000 mm in the main grain-producing areas, creating favorable conditions for agriculture, but with limited per capita arable land (Liu et al., 2018a; Pan and Gao, 2023; Sun et al., 2019). Therefore, the development of diversified multi-cropping systems in the Yangtze River Basin is a viable strategy for increasing crop yields and farmers’ incomes (Li et al., 2023a). The multi-cropping system involves the cultivation of multiple crops on the same land or intercropping of different crops within the same area in a year, exemplifying temporal and spatial intensification (Waha et al., 2020). However, as the multiple-cropping index increases, there is a disconnect between high yield and high efficiency (Tao et al., 2013). The escalated use of agricultural inputs on finite arable lands will result in increased environmental costs for crop production (Li et al., 2023b). Therefore, designing efficient and sustainable multiple-cropping patterns is crucial for agricultural sustainability in the Yangtze River Valley of China.

Based on the complexity of agroecosystems, Odum H T, a famous American ecologist, put forward emergy analysis theory in the 1980s to comprehensively analyze agroecosystems. Emergy assessment can take into account aspects that are not usually addressed in traditional energy assessments, such as ecosystem services. In other words, emergy analysis links natural ecosystems to human-led economic systems by providing “supply-side” assessments (Houshyar et al., 2018). Emergy is defined as the total amount of available energy used directly or indirectly to create a product or service (Odum, 1996). Emergy theory translates the matter, energy, and value within the agricultural eco-economic system into a uniform metric of emergy, equating it to solar energy, for measuring and comparing the true value of different ecosystem components. This method has been extensively applied to assess agricultural systems across various scales and management approaches, examining their resource use, productivity, environmental impacts, and sustainability (Zhang et al., 2016), including national agricultural systems (Chen et al., 2006; Gasparatos, 2011; Ghisellini et al., 2014; Hu et al., 2022), regional agricultural systems (Amini et al., 2020; Ferreyra, 2006; Kazemi et al., 2015; Zadehdabagh et al., 2022), and specific planting systems (Fan et al., 2018; Ghaley and Porter, 2013; Sha et al., 2015; Wu et al., 2013; Zhang et al., 2012).

The emergy analysis method has been utilized to assess the ecological benefits of diverse agricultural cultivation patterns. Xu et al. (2019) assessed the environmental and economic advantages of rice monoculture, traditional rice–crab system, and optimized rice–crab practices in the Liaohe River Basin. It was proposed that the optimized rice–crab system enhanced the sustainability and economic viability of rice production. Li et al. (2018) used the emergy methodology to appraise the sustainability of three rice cropping systems in Jiangsu Province. The results illustrated that labor inputs were the main cost of rice cropping systems, while feed input held greater significance in the rice–fish and rice–duck systems. Chen et al. (2021a) conducted a comparative analysis of the sustainability of rice–wheat, rice–fallow, and rice–crayfish rotation systems using the emergy assessment method. They pointed out chemical fertilizers, diesel fuel, water, electricity, and feed as key contributors to the rice–crayfish rotation system’s high environmental impact and low sustainability. Li et al. (2023b) analyzed the emergy benefits of seven distinct multiple-cropping systems in South China, using double-cropping rice as a benchmark. They concluded that the corn-rice/ryegrass × cowpea mixed model surpassed other planting models in efficiency and sustainability. Li et al. (2021) examined the economic benefits and sustainability of various cultivation patterns in hilly regions of southwest China and indicated that the novel triple-cropping system markedly enhanced economic outcomes and environmental sustainability relative to both the traditional double- and triple-cropping systems. Cui et al. (2018) compared the wheat–corn rotation system with the monoculture corn system in North China, integrating economic analysis, emergy assessment, and life cycle assessment. They found that the monoculture corn system posed minimal environmental impact and exhibited high sustainability. Therefore, it is important to study the efficiency and sustainability of different planting patterns, explore the balance of economic and environmental benefits, and provide decision-making support for agricultural production.

The Yangtze River Basin is rich in dryland resources, and corn is the main food crop, which plays an important role in ensuring food security in China (Wang et al., 2023). As a C4 crop with high adaptability and efficient utilization of light, temperature, water, fertilizer, and other resources, corn plays a connecting role in multiple-cropping systems (Guo et al., 2023b; Li et al., 2020). Previous studies have indicated that enhancing biodiversity spatially or temporally of cropping systems may promote ecosystem services, including pest and disease control, soil fertility, and carbon sequestration. Diversification through the inclusion of legumes in cropping systems represents a key strategy for sustainable agriculture (Zhao et al., 2022). In recent years, the emergy analysis of cultivation patterns mostly focused on the paddy field ecosystem, while the research on multiple-cropping patterns in the dryland ecosystem was relatively few.

Therefore, this study was based on the climatic characteristics and grain demand characteristics of the Yangtze River Basin, which is characterized by insufficient triple cropping, excessive double cropping, cold temperatures in late spring, and high temperatures in summer. Field studies were carried out in Sichuan, Hubei (upper Yangtze River), Hunan, Jiangxi (middle Yangtze River), and Jiangsu (lower Yangtze River) to establish a variety of dryland replanting patterns centered on a maize–soybean strip intercropping system. Based on the field experiments, the emergy assessment method was introduced, and the efficiency and sustainability of multiple-cropping patterns in various regions of the Yangtze River Basin were quantitatively compared with those of the double-cropping system in order to achieve the sustainable goals of high yield of dryland crops, efficient resource utilization, and balance between utilization and nutrition. This study would provide valuable information for developing sustainable multi-cropping systems in the Yangtze River Basin.

## 2 Material and methods

### 2.1 Experimental site and design

Maize is the third largest food crop in southern China. Rape, potato, broad bean, and soybean are popular crops in the study area. Forage rape and ryegrass could effectively improve soil fertility and realize the combination of use and nutrition. In addition, soybeans are a core crop for new agriculture in South China. The interplanting of soybean and maize could coexist harmoniously with local main grain crops such as rice and corn and avoid the competition between crops in land, time, and space. Taking the traditional double-cropping system as the control (CK), aiming at the climatic conditions and diversified market demand, diversified multiple-cropping systems based on corn were designed in five locations in the Yangtze River Basin. Generally speaking, we try to establish a diversified multiple-cropping system in which grain, cash crops, and feed are coordinated: changing single cropping into intercropping, changing double cropping into triple cropping, and changing grain crops into cash crops and feed crops. Increasing crop yields and resource utilization rates would help to achieve the sustainable goal of balanced utilization and nutrition of the Yangtze River Basin, guaranteeing food security and increasing farmers’ incomes.

**Figure 1:**
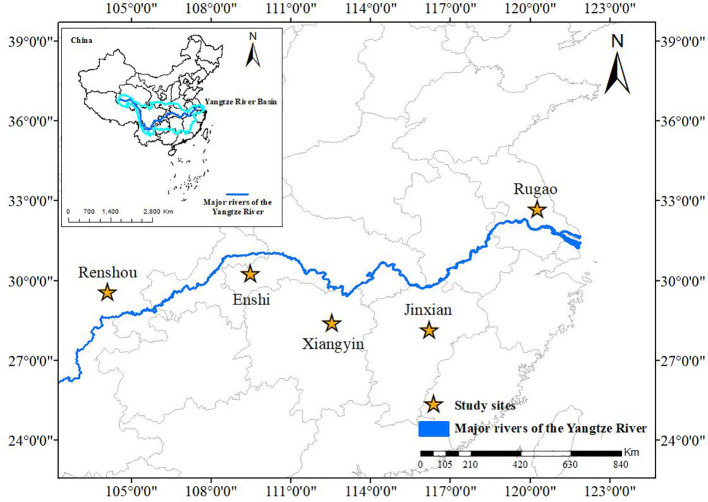
Location of the study area and Yangtze River Basin.

The experimental sites were located in five representative locations in the Yangtze River Basin. The experiment in Sichuan (upper reaches of the Yangtze River) was carried out from Oct. 2018 to Nov. 2020, including two complete planting cycles. The experiments in Hubei (upper reaches of the Yangtze River), Jiangxi, Hunan (middle reaches of the Yangtze River), and Jiangsu (lower reaches of the Yangtze River) were carried out from Octo. 2016 to Nov. 2020, including four complete planting cycles. The location of the study site is shown in **Figure 1**. The study sites were located at Renshou County, Meishan City, Sichuan Province (104°08′E, 29°59′N); Jinxian County, Nanchang City, Jiangxi Province (116°20′E, 28°15′N); Xiheba County, Enshi City, Hubei Province (109°47′E, 30°29′N); Xiangyin County, Yueyang City, Hunan Province (112°53′E, 28°43′N); and Rugao County, Nantong City, Jiangsu Province (120°27′E, 32°70′N). Meteorological data were sourced from local meteorological stations and included solar radiation, daily temperatures, hours of sunshine, and rainfall levels. The initial soil properties of the research field are shown in **Table 1**. The single-factor randomized block design was adopted in the experiment, with 19 treatments repeated three times. The plot area was 46.2 m^2^ (6.6 m × 7 m) in Sichuan, 35 m^2^ (5 m × 7 m) in Jiangxi, and 30 m^2^ (4 m × 7.5 m) in Jiangsu, Hunan, and Hubei. The main local varieties were selected as experimental varieties. The specific varieties, densities, and fertilization rates are shown in **Table 2**. The specific experimental design, sowing date, and harvest date of crops with different multiple-cropping patterns are shown in **Table 3**.

**Table 1:** Basic information on experimental sites in different regions of the Yangtze River Basin.

Test plot	Renshou, Sichuan	Enshi, Hubei	Xiangyin, Hunan	Jinxian, Jiangxi	Rugao, Jiangsu
Average annual temperature (°C)	16–18	15–17	15–17	17–18	14–16
Frost-free season (d)	230–340	230–300	253–311	240–307	256–326
Year-round sunshine (h)	1,000–1,400	1,400–2,200	1,400–2,200	1,400–2,200	2,200–3,000
Average annual rainfall (mm)	622.0	1,355.5	1,199.8	1,737.0	1,478.1
Elevation (m)	431	900	500	25	6
pH	6.8	6.3	5.1	5.0	7.4
organic matter (g kg^−1^)	20.52	15.02	22.80	20.70	12.27
Total N (g kg^−1^)	1.06	1.04	1.22	0.93	0.88
Total P (g kg^−1^)	0.47	1.08	0.68	0.42	0.81
Total K (g kg^−1^)	9.78	17.76	11.80	27.60	11.81
Available N (mg kg^−1^)	110.00	125.73	125.40	80.79	101.03
Available P (mg kg^−1^)	12.60	19.81	7.13	14.90	10.40
Available K (mg kg^−1^)	115.00	158.80	75.30	114.00	51.84

**Table 2:** Date of sowing and harvest of crops with multi-cropping systems in Yangtze River Basin.

Test site	Cropping pattern	1st mature		2nd mature		3rd mature	
		Sowing date	Harvest date	Sowing date	Harvest date	Sowing date	Harvest date
Renshou, Sichuan	R1: wheat–corn	Early Nov.	Mid May	Mid May	Mid Sep.		
	R2: wheat–corn//soybean	Early Nov.	Mid May	Mid May	Mid Sep.	Early Jun.	Late Oct.
	R3: potato–corn/soybean	Early Dec.	Mid Apr.	Late Apr.	Mid Aug.	Early Jun.	Late Oct.
Enshi, Hubei	E1: forage rape–corn	Late Oct.	Mid Apr.	Late Apr.	Early Sep.		
	E2: forage rape–corn//soybean	Late Oct.	Mid Apr.	Late Apr.	Early Sep.	Late Apr.	Late Aug.
	E3: rape–corn//soybean	Late Oct.	Mid May	Early Jun.	Early Sep.	Early Jul.	Early Jan.
	E4: potatoes/corn/soybean	Late Dec.	Early Jun.	Early Apr.	Early Sep.	Late Jul.	Early Jan.
Xiangyin, Hunan	X1: rape–corn	Early Oct.	Early May	Early Jun.	Early Sep.		
	X2: rape–corn//soybean	Early Oct.	Early May	Early Jun.	Late Sep.	Late May	Late Sep.
	X3: forage rape–corn/soybean	Mid Nov.	Late Mar.	Early Apr.	Late Jul.	Late May	Late Sep.
	X4: forage rape–corn–soybean	Mid Nov.	Mid Mar.	Early Apr.	Late Jul.	Early Aug.	Early Jan.
Jinxian, Jiangxi	N1: winter leisure–corn//soybean			Early Apr.	Late Jul.	Early Apr.	Mid Jul.
	N2: potatoes/corn/soybean	Mid Nov.	Early May	Early Apr.	Jul. Late	Late May	Late Aug.
	N3: ryegrass–corn/soybean	Early Oct.	Mid Mar.	Early Apr.	Jul. Late	Mid May	Early Aug.
Rugao, Jiangsu	F1: fresh faba bean–fresh corn	Mid Nov.	Late May	Mid Jun.	Aug. Late		
	F2: fresh faba bean/fresh corn-fresh soybean	Mid Nov.	Late May	Early Apr.	Jul. Mid	Late Jul.	Late Oct.
	F3: fresh faba bean/fresh corn-fresh corn	Mid Nov.	Late May	Early Apr.	Jul. Mid	Late Jul.	Mid Oct.
	F4: wheat–fresh corn/fresh soybean	Mid Nov.	Late May	Mid Jun.	Aug. Late	Late Jul.	Late Oct.
	F5: wheat–fresh corn/fresh corn	Mid Nov.	Late May	Mid Jun.	Aug. Late	Late Jul.	Mid Oct.

/, relay strip intercropping; //, strip intercropping.

**Table 3:** Detailed information on agricultural practices of the different crops in the experiment.

Test site	Cropping pattern	1st mature			2nd mature			3rd mature		
		Variety	Density (kg hm^−2^)	Fertilizer N:P_2_O_5_:K_2_O (kg hm^−2^)	Variety	Density (kg hm^−2^)	Fertilizer N:P_2_O_5_:K_2_O (kg hm^−2^)	Variety	Density (kg hm^−2^)	Fertilizer N:P_2_O_5_:K_2_O (kg hm^−2^)
Renshou, Sichuan	R1	Shumai 969	240.0	150.0:90.0:90.0	Rongyu 1210	6.0	225.0:75.0:75.0			
	R2	Shumai 969	240.0	150.0:90.0:90.0		6.0	225.0:75.0:75.0	Nandou 25	15	0:45.0:0
	R3	Favorita	10.0	150.0:30.0:150.0		6.0	225.0:75.0:75.0		15	0:45.0:0
Enshi, Hubei	E1	Dadi 199	4.5	12.5:12.5:12.5	Qingqing 500	4.5	43.0:26.0:14.0			
	E2		4.5	12.5:12.5:12.5		4.5	43.0:26.0:14.0	05-48	12	4.5:4.5:4.5
	E3		4.5	12.5:12.5:12.5		4.5	43.0:26.0:14.0	Shiyuehuang	12	4.5:4.5:4.5
	E4	Emalingshu 10	5.5	46.5:29.5:17.5		4.5	43.0:26.0:14.0		12	4.5:4.5:4.5
Xiangyin, Hunan	X1	Fengyou 823	30.0	159.0:202.5:90.0	Denghai 605	6.0	228.0:202.5:90.0			
	X2	Fengyou 823	30.0	159.0:202.5:90.0		6.0	228.0:202.5:90.0	Zhongdou 41	16.5	68.3:146.3:33.8
	X3	Youfei 1	15.0	34.5:0:0		6.0	228.0:202.5:90.0		16.5	68.3:146.3:33.8
	X4	Youfei 1	15.0	34.5:0:0		6.0	228.0:202.5:90.0	Xiangchundou 24	16.5	68.3:146.3:33.8
Jinxian, Jiangxi	N1				Jixiang 1	6.0	180.0:72.0:90.0	Handou 1	15	34.5:72.0:90.0
	N2	Tianyuan chun	10.0	112.5:112.5:112.5		6.0	180.0:72.0:90.0	Nandou 12	15	34.5:72.0:90.0
	N3	Xindaye	850.0	0:0:0		6.0	180.0:72.0:90.0		15	34.5:72.0:90.0
Rugao, Jiangsu	F1	Tongcanxian 7	7.0	56.5:0:0	Suyunuo 14	6.0	146.5:90.0:90.0			
	F2	Tongcanxian 7	7.0	56.5:0:0		60	146.5:90.0:90.0	Tongxian 6	15	22.5:0:0
	F3	Tongcanxian 7	7.0	56.5:0:0		6.0	146.5:90.0:90.0	Suyunuo 14	6	146.5:90.0:90.0
	F4	Youngmai 16	260.0	54.5:30.0:30.0		6.0	146.5:90.0:90.0	Tongxian 6	15	22.5:0:0
	F5	Youngmai 16	260.0	54.5:30.0:30.0		6.0	146.5:90.0:90.0	Suyunuo 14	6	146.5:90.0:90.0

### 2.2 Emergy assessments

#### 2.2.1 Emergy flow description

**Figure 2:**
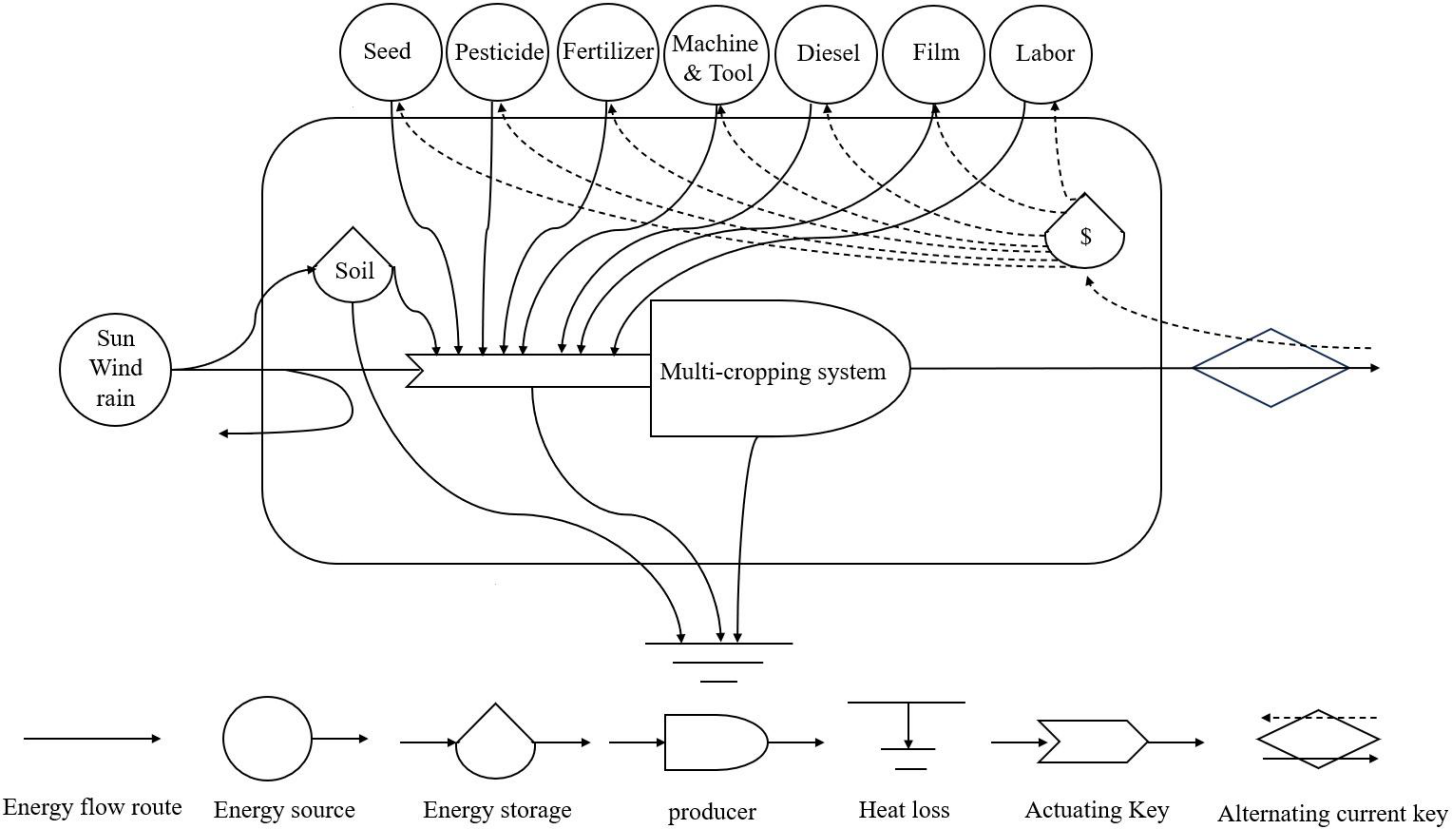
Energy flow diagram of multi-cropping agricultural systems in the Yangtze River Basin.

Based on the standard emergy theory proposed by Odum (1996), the energy flow diagram of the multi-cropping agricultural system is shown in **Figure 2**. The planting system needs 1) renewable natural resources (R), including sunlight, wind, and rain; 2) non-renewable natural resources (NR), such as topsoil loss; and 3) economically purchased resources (P) including fertilizers, pesticides, films, machinery, diesel oil, seeds, and labor. According to the renewable factor (RNF), economically purchased resources (P) were divided into purchased renewable resources (PR) and non-renewable resources purchased (PN). The RNFs used in the present case were mainly derived from previously published papers (Chen et al., 2021b; Li et al., 2023b; Xu et al., 2019; Wang et al., 2017). To compare the sustainability of different planting methods under the same standard, all inputs were multiplied by the corresponding unit energy value (UEV) and converted into a unified energy flow (Li et al., 2023b). The UEVs used in this study were screened according to the actual situation in China, mainly from previously published papers (Chen et al., 2021b; Houshyar et al., 2018; Jiang et al., 2007; Li et al., 2023a, 2023b; Moonilall et al., 2020; Xu et al., 2019). The global energy baseline adopted was 12.00 × 10^24^ seJ yr^−1^ (Brown et al., 2016).

#### 2.2.2 Emergy-based indices

In this study, emergy-based indices were selected—unit emergy value (UEV), unit non-renewable value (UNV), emergy yield ratio (EYR), environmental loading ratio (ELR), and emergy sustainability index (ESI)—to compare the efficiency and sustainability of different cultivation patterns. In emergy assessments, among the inputs of solar energy, rainwater, and wind energy, rainwater and wind energy were regarded as by-products of the sun. In order to avoid double calculation, only the maximum energy values of the three were calculated. The description and expression of each emergy index are shown in **Table 4**.

**Table 4:** Indicator description based on emergy.

Indicator	Expression	Meaning
Unit emergy value (UEV)	T/Y	Evaluate the production efficiency of the system.
Unit non-renewable value (UNV)	(NR + PN)/Y	Measure the utilization rate of non-renewable resources under unit output.
Emergy yield ratio (EYR)	T/(PN + PR)	The ability of the system to utilize local resources in external input.
Environmental loading ratio (ELR)	(NR + PN)/(R + PR)	Evaluate the pressure caused by the system on the local environment.
Emergy sustainability index (ESI)	EYR/ELR	Evaluate the sustainability of the system.

R, renewable natural resources; NR, non-renewable natural resources; PN, non-renewable resources purchased; PR, purchased renewable resources; T, emergy total input; Y, total energy output.

#### 2.2.3 Data sources

In this study, the raw data of agricultural inputs were mainly derived from the experimental recording. Each treatment was calculated in the unit area of 1 hm^2^. The consumed amounts, types, and application methods of the agricultural inputs were recorded in as much detail as possible to improve the accuracy of the data. Meteorological data were collected from the automatic weather station in the experimental field. The yields of different crops were collected and measured during the harvest, specifically, wheat, rape, ryegrass, and potato; the actual harvest yield was converted into hectare yield. Forage rape: Biomass was counted as yield. Spring maize, summer maize, spring soybeans, and summer soybeans: in the maize maturity stage, the harvested maize was threshed and dried as the actual output, and in the soybean maturity stage, the harvested soybeans were dehulled and dried as the actual output. Fresh broad bean, fresh maize, and fresh soybean: the yield of fresh broad bean and fresh soybean was calculated by fresh silkworm pod and fresh big pod, and the yield of fresh maize was calculated by fresh ear. The annual productivity of the cropping system was the sum of all yield emergy of each crop product. Moreover, according to the crop yields of different crops, the economic output was estimated according to the corresponding price: wheat at 0.29 $ kg^−1^, forage rape at 0.03 $ kg^−1^, oilseed rape at 0.80 $ kg^−1^, potato at 0.17 $ kg^−1^ (Li et al., 2023), ryegrass at 0.09 $ kg^−1^, soybean at 0.95 $ kg^−1^, feed maize at 0.32 $ kg^−1^, sweet maize at 0.43 $ kg^−1^ (Xian et al., 2023), fresh faba bean at 0.15 $ kg^−1^, and fresh soybean at 0.58 $ kg^−1^ (local market survey data). According to data from the National Bureau of Statistics, this study used the average exchange rate of 6.8974 Chinese Yuan per US dollar in 2020 (Xian et al., 2023). The seed yield of maize was calculated with 14% moisture. The seed yield of wheat and soybean was calculated with 13% moisture. The rapeseed yield was calculated with 11% moisture.

## 3 Results

### 3.1 Emergy inputs

The total emergy input (T) of the double-cropping control in five locations was as follows: 1.62E+16 (R1), 1.79E+16 (E1), 1.74E+16 (X1), 1.22E+16 (N1) and 1.95E+16 (F1) (**Table 5**; **Supplementary Table S1**). The T of triple-cropping systems in different places increased by 26.21% (Sichuan), 36.65% (Hubei), 6.87% (Hunan), 51.17% (Jiangxi), and 19.41% (Jiangsu) compared to the control. Consequently, the triple-cropping system consumes more resources than the traditional double-cropping system. From the perspective of sources, the emergy flows from labor and chemical fertilizer made up the top two contributors to the treatments, with labor input contribution rates of 57.12%–59.93% (Sichuan), 82.54%–87.20% (Hubei), 48.23%–52.61% (Hunan), 51.33%–57.58% (Jiangxi), and 71.32%–78.07% (Jiangsu). The contribution rate of fertilizer input was 17.00%–23.39% (Sichuan), 3.08%–3.66% (Hubei), 26.34%–33.37% (Hunan), 18.16%–21.74% (Jiangxi), and 10.21%–15.82% (Jiangsu). In summary, the triple-cropping system consumed more resources than the traditional double-cropping system, and controlling labor and fertilizer input was the key to optimizing the multi-cropping system.

**Table 5:** Aggregated emergy flows of different multi-cropping systems in the study.

Test site	Cropping pattern	Solar emergy flows (sej ha^−1^ yr^−1^)				
		Renewable natural inputs (R)	Non-renewable natural inputs (NR)	Non-renewable resources purchased (PN)	Purchased renewable resources (PR)	Total emergy input (T)
Sichuan	R1	2.25E+14	8.01E+14	1.34E+16	1.77E+15	1.62E+16
	R2	2.25E+14	8.01E+14	1.54E+16	2.09E+15	1.85E+16
	R3	2.25E+14	8.01E+14	1.64E+16	4.83E+15	2.23E+16
Hubei	E1	4.67E+14	5.86E+14	1.50E+16	1.86E+15	1.79E+16
	E2	4.67E+14	5.86E+14	1.81E+16	2.33E+15	2.15E+16
	E3	4.67E+14	5.86E+14	1.99E+16	2.56E+15	2.35E+16
	E4	4.67E+14	5.86E+14	2.30E+16	4.44E+15	2.85E+16
Hunan	X1	4.16E+14	8.90E+14	1.50E+16	1.09E+15	1.74E+16
	X2	4.16E+14	8.90E+14	1.80E+16	1.39E+15	2.07E+16
	X3	4.16E+14	8.90E+14	1.50E+16	1.26E+15	1.76E+16
	X4	4.16E+14	8.90E+14	1.50E+16	1.26E+15	1.76E+16
Jiangxi	N1	5.90E+14	8.08E+14	9.19E+15	9.07E+14	1.15E+16
	N2	5.90E+14	8.08E+14	1.54E+16	4.14E+15	2.09E+16
	N3	5.90E+14	8.08E+14	1.11E+16	1.20E+15	1.37E+16
Jiangsu	F1	5.12E+14	4.80E+14	1.61E+16	2.44E+15	1.95E+16
	F2	5.12E+14	4.80E+14	1.78E+16	2.73E+15	2.15E+16
	F3	5.12E+14	4.80E+14	2.46E+16	3.39E+15	2.90E+16
	F4	5.12E+14	4.80E+14	1.43E+16	2.39E+15	1.76E+16
	F5	5.12E+14	4.80E+14	2.11E+16	3.05E+15	2.52E+16

### 3.2 Emergy input structure

As shown in **Table 5** and **Supplementary Table S1**, the total renewable energy input (R + PR) of double-cropping systems in each region was as follows: 2.00E+15 (R1), 2.33E+15 (E1), 1.51E+15 (X1), 2.17E+15 (N1), and 2.95 E+15 (F1). The total renewable energy input (R + PR) of triple-cropping systems in different regions increased by 84.43% (Sichuan), 53.62% (Hubei), 13.72% (Hunan), 100.68% (Jiangxi), and 15.46% (Jiangsu) compared to the control. Labor (renewable) was the largest emergy input in the total renewable emergy input (R + PR). The total non-renewable energy input (NR + PN) of the double-cropping system in each region was 1.42E+16 (R1), 1.56E+16 (E1), 1.59E+16 (X1), 1.00E+16 (N1), and 1.66E+16 (F1). The total non-renewable energy input (NR + PN) of triple-cropping systems in different regions increased by 18.00% (Sichuan), 34.11% (Hubei), 6.22% (Hunan), 40.43% (Jiangxi), and 20.11% (Jiangsu) compared to the control. In the total non-renewable emergy input (NR + PN), the top two emergy inputs were labor (non-renewable) and fertilizer. To sum up, the triple-cropping system increased the dependence on the labor force on renewable and non-renewable emergy input compared with the traditional double-cropping system.

### 3.3 Emergy yields

**Figure 3:**
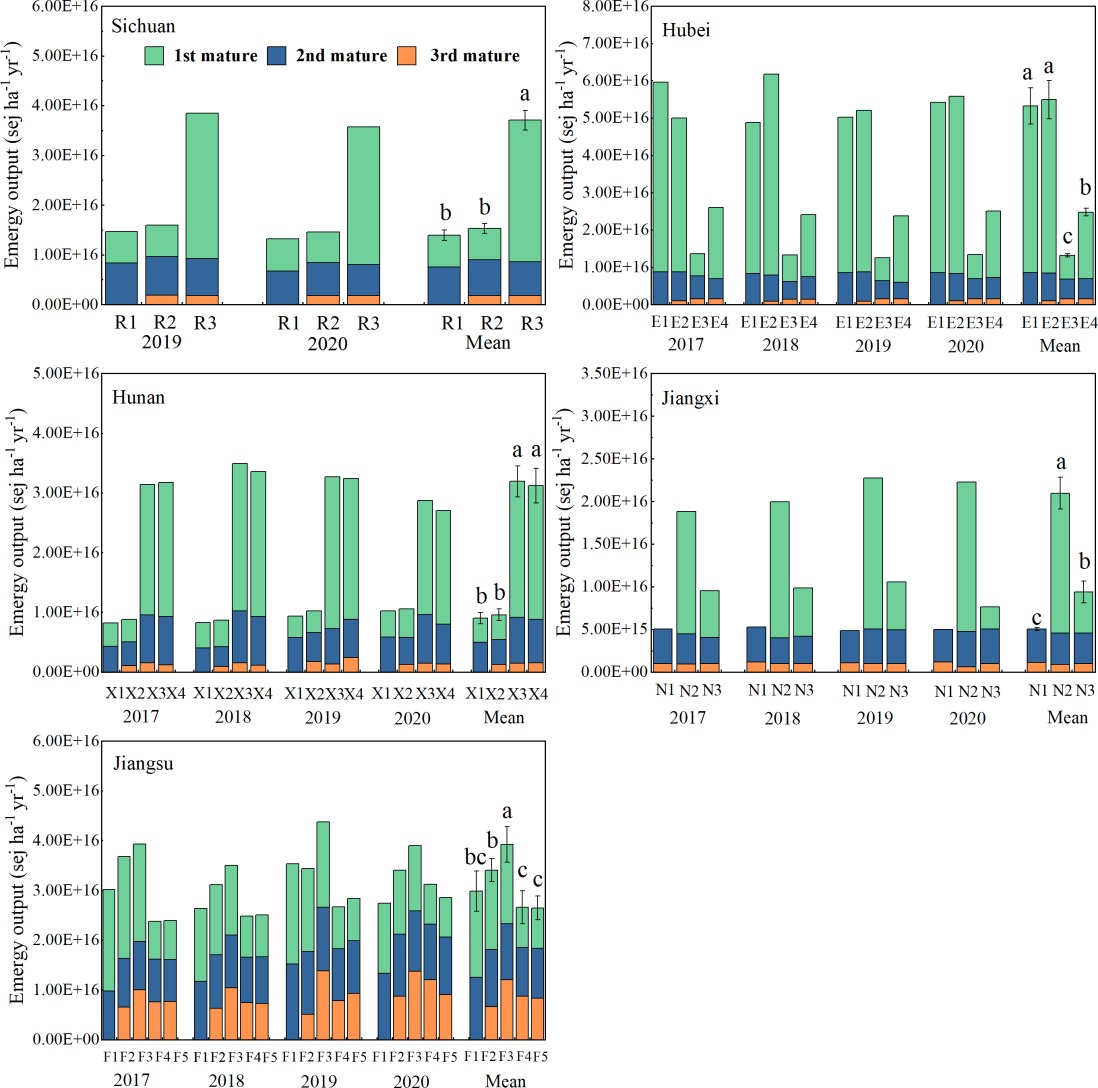
Emergy output of multi-cropping agricultural systems in the Yangtze River Basin. Different lowercase letters indicate significant differences among treatments (p < 0.05).

As shown in **Figure 3**, the annual emergy output of R2 and R3 increased by 8.60%–161.96% on average compared with R1. The annual emergy output of E2 increased by 3.06%, while E3 and E4 decreased by 75.15% and 53.49% compared with E1. The output of X1, X2, X3, and X4 increased by 6.08%–254.09% on average. The output of N1, N2, and N3 increased by 86.23%–315.75% on average. The annual emergy output of F2 and F3 increased by 14.25% and 31.59%, while that of F4 and F5 decreased by 10.24% and 11.84%, respectively, compared with F1. The results illustrated that the annual productivity of the triple-cropping system was improved compared with that of the double-cropping system, and the selection of winter crops was very important for the improvement of annual productivity. Choosing potato or forage rape in the middle and upper reaches of the Yangtze River and fresh broad bean in the lower reaches of the Yangtze River could significantly improve the emergy output.

### 3.4 Economic benefit

**Figure 4:**
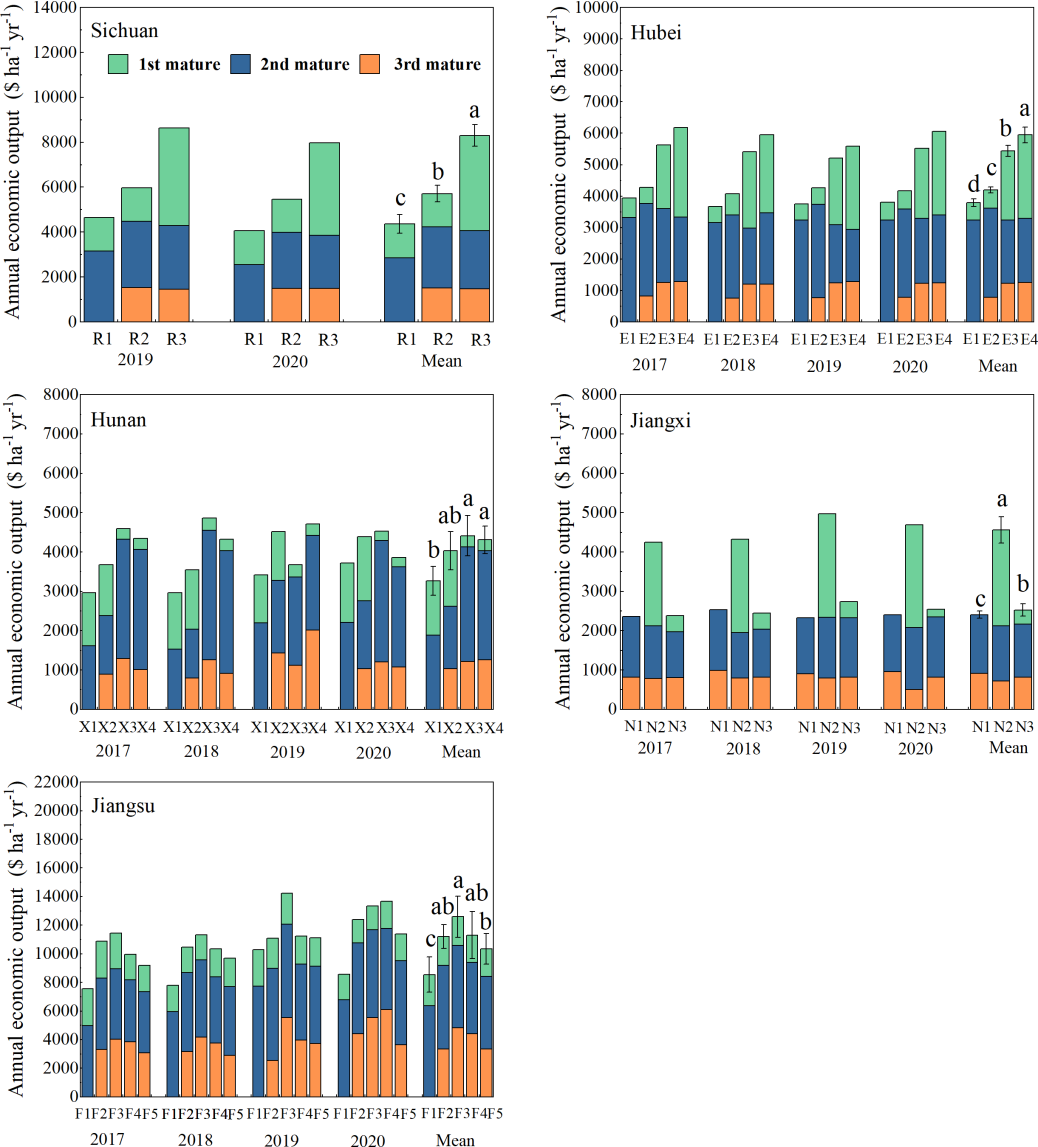
Annual economic output of multi-cropping agricultural systems in the Yangtze River Basin. Different lowercase letters indicate significant differences among treatments (p < 0.05).

As shown in **Figure 4**, the annual economic outputs of R2 and R3 increased by 30.91% and 90.23%, respectively, compared with the control R1, reaching a significant level. The annual economic outputs of E2, E3, and E4 were significantly higher than those of E1, increasing by 10.61%, 43.39%, and 56.72%, respectively. Compared with those of N1, the annual economic outputs of N2 and N3 increased by 89.42% and 5.01%, respectively. Compared with X1, the annual economic outputs of X2, X3, and X4 increased by 23.46%, 35.37%, and 32.19%, respectively. The annual economic outputs of F2, F3, F4, and F5 increased by 31.37%, 47.82%, 32.83%, and 21.02%, respectively, reaching a significant level. Therefore, due to the high economic value of soybeans, increasing soybeans in the planting system has increased the annual economic output by 10.61%–90.23%, which was conducive to increasing the willingness of farmers to plant.

### 3.5 Emergy-based efficiency

#### 3.5.1 Unit emergy value

**Figure 5:**
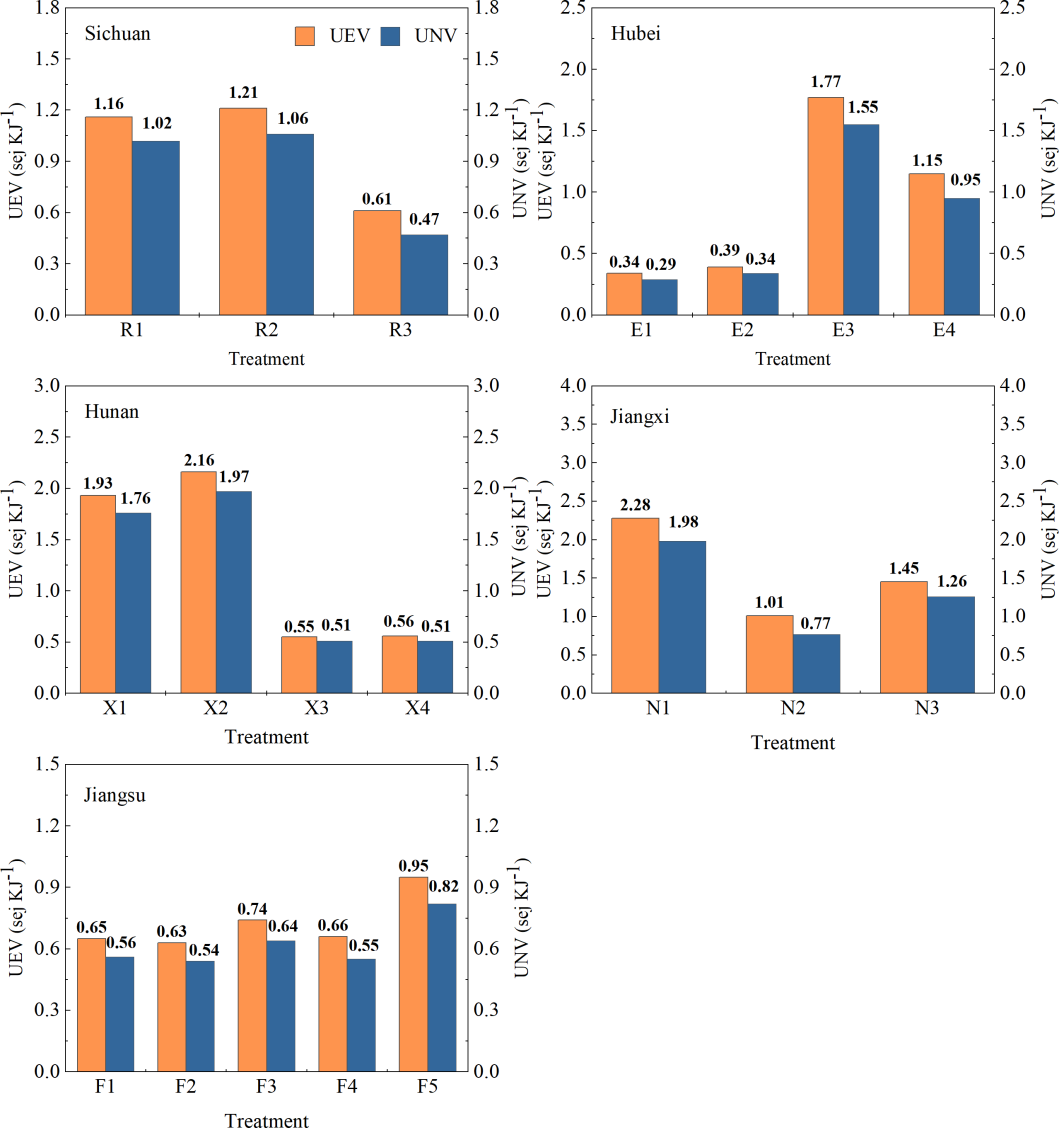
Efficiency assessments of multi-cropping agricultural systems in the Yangtze River Basin.

The UEVs of double cropping in each region were as follows: 1.16 (R1), 0.34 (E1), 1.93 (X1), 2.28 (N1), and 0.65 (F1) (**Figure 5**; **Table 6**). The UEV of R2 increased by 4.30%, while that of R3 decreased by 48.09% compared with R1. Compared with the control E1, the UEVs of E2, E3, and E4 increased by 16.22%–467.25%. The UEV of X2 increased by 12.14%, and X3 and X4 decreased by 71.49% and 70.84%, respectively, compared with X1. The UEVs of N2 and N3 decreased by 56.17% and 36.23%, respectively, compared with N1. The UEVs of F3, F4, and F5 increased by 12.86%, 1.21%, and 45.15%, respectively, while the UEV of F2 decreased by 3.74% compared with F1. Consequently, with increased emergy input, the emergy utilization rate of the triple-cropping system in each region was lower than that of the double-cropping system. Moreover, the total input emergy efficiency of R3, X3, X4, N2, N3, and F2 was higher than that of other cultivation patterns within the same region.

**Table 6:** The difference in emergy index between diversified multi-cropping systems and the control in the same experimental site.

Test site	Cropping pattern	UEV	UNV	EYR	ELR	ESI
Sichuan	R2	4.30%	4.13%	−0.84%	−1.29%	0.00%
	R3	−48.09%	−54.20%	−1.83%	−51.89%	104.03%
Hubei	E2	16.22%	16.20%	−1.03%	−0.11%	0.00%
	E3	427.55%	428.11%	−1.46%	0.82%	−2.26%
	E4	241.71%	225.09%	−2.26%	−28.23%	36.17%
Hunan	X2	12.14%	12.10%	−1.28%	−0.41%	0.00%
	X3	−71.49%	−71.79%	0.00%	−9.93%	10.95%
	X4	−70.84%	−71.12%	0.00%	−9.93%	10.95%
Jiangxi	N2	−56.17%	−60.99%	−5.88%	−48.68%	83.39%
	N3	−36.23%	−36.28%	−2.14%	−0.60%	0.00%
Jiangsu	F2	−3.74%	−3.73%	−0.48%	0.00%	0.00%
	F3	12.86%	15.04%	−1.72%	14.29%	−14.01%
	F4	1.21%	−0.42%	0.58%	−9.78%	11.48%
	F5	45.15%	46.72%	−1.18%	7.64%	−8.20%

UEV, unit emergy value; UNV, unit non-renewable value; EYR, emergy yield ratio; ELR, environmental loading ratio; ESI, emergy sustainability index.

#### 3.5.2 Unit non-renewable value

As presented in **Figure 5** and **Table 6**, the values and trends of UNVs were similar to those of UEVs. The UNV of R2 showed a 4.13% increase, and that of R3 showed a decrease of 54.20%, relative to R1. Relative to those of E1, the UNVs of E2, E3, and E4 showed an increase ranging from 16.20% to 428.11%. The UNV of X2 increased by 12.10%, while the UNVs of X3 and X4 decreased by 71.79% and 71.12%, respectively, compared to X1. The UNVs of N2 and N3 decreased by 60.99% and 36.28%, respectively, relative to N1. The UNVs of F3 and F5 increased by 15.04% and 46.72%, respectively, while those of F2 and F4 decreased by 3.73% and 0.42%, respectively, compared to F1. The results indicated that with the same winter crops, the triple-cropping system exhibited a lower non-renewable resource utilization rate compared to the double-cropping system.

### 3.6 Emergy-based sustainability

#### 3.6.1 Emergy yield ratio

**Figure 6:**
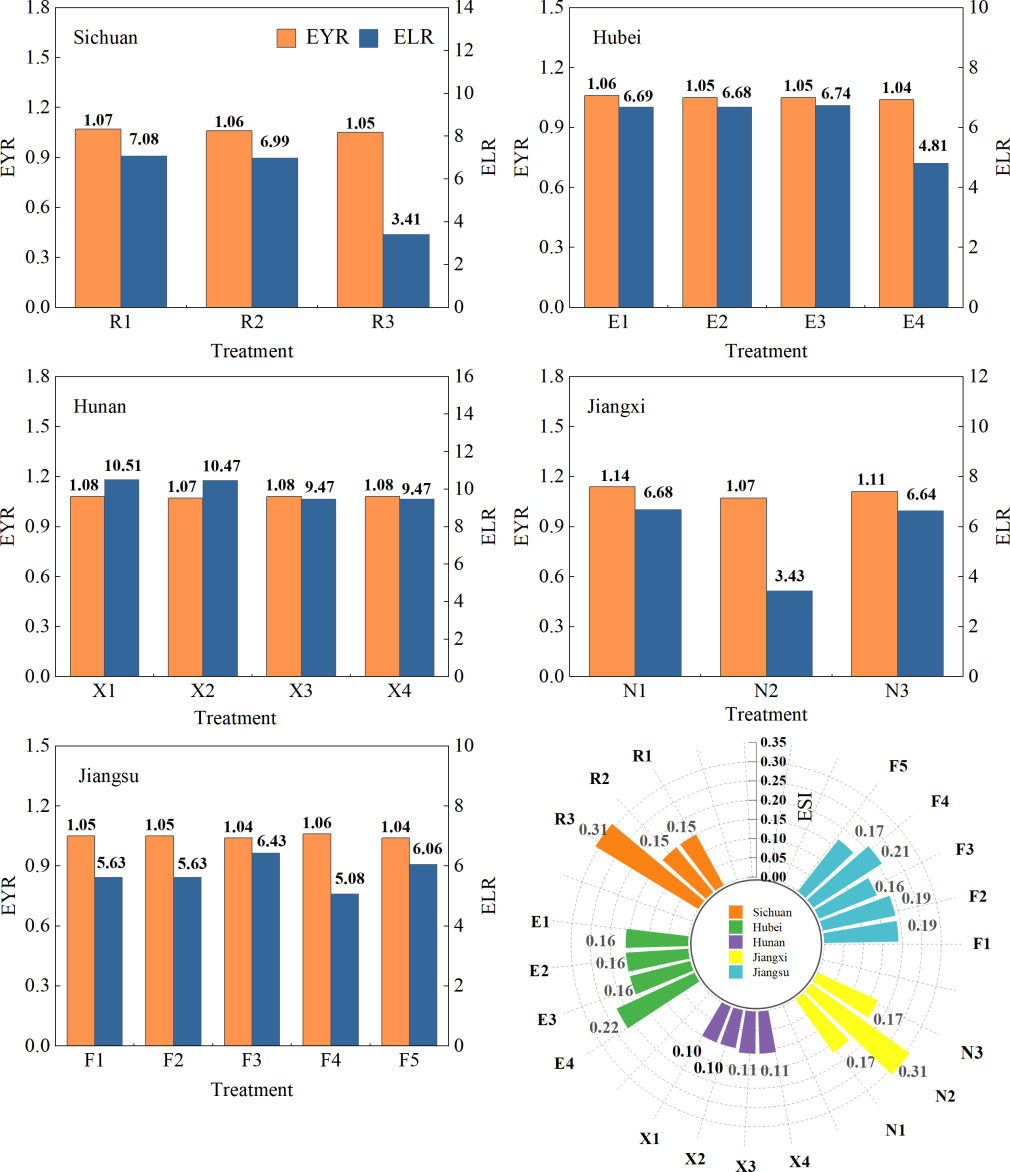
Environmental sustainability assessments of multi-cropping agricultural systems in the Yangtze River Basin.

As shown in **Figure 6** and **Table 6**, the EYRs of R2 and R3 decreased by 0.84% and 1.83%, respectively, compared with R1. Compared with the EYR of E1, the EYRs of all treatments in Hubei decreased by 1.03%–2.26%. The EYR of X2 decreased by 1.28% compared with X1, and the EYRs of X3 and X4 were the same as the EYR of X1. The EYRs of the triple-cropping system in Jiangxi decreased by 2.14% and 5.88% compared with N1. The EYRs of F2, F3, and F4 decreased by 0.48%, 1.72%, and 1.18%, respectively, while the EYR of F5 increased by 0.58% compared with F1. The results showed that the triple-cropping system reduces the dependence on natural resources compared with the double-cropping system.

#### 3.6.2 Environmental loading ratio

The ELRs of double-cropping systems in different regions were as follows: 7.08 (R1), 6.69 (E1), 10.51 (X1), 6.68 (N1), and 6.53(F1) (**Figure 6**; **Table 6**). Compared to the ELR of R1, the ELRs of R2 and R3 decreased by 1.29% and 51.89%, respectively. The ELRs of E2 and E4 decreased by 0.11% and 28.23%, respectively, and the ELR of E3 increased by 0.82% relative to E1. Relative to the ELR of X1, the ELRs of X2, X3, and X4 decreased within the range of 0.41%–9.93%. Compared to the ELR of N1, the ELRs of N2 and N3 decreased by 48.68% and 0.60%, respectively. Relative to that of F1, the ELR for F4 decreased by 9.78%, that of F2 remained unchanged, and the ELRs of F3 and F5 increased by 14.24% and 7.64%, respectively. The results indicate that the triple-cropping system alleviates environmental pressure more effectively than the double-cropping system due to its efficient resource utilization.

#### 3.6.3 Emergy sustainability index

As presented in **Figure 6** and **Table 6**, the ESI of R3 increased by 104.03%, and the ESI of R2 was as same as that of R1. The ESI of E4 increased by 36.17%, and that of E3 decreased by 2.26% compared with E1; E2 had the same ESI as E1. The ESI of X3 and X4 increased by 10.95% compared with X1, and the ESI of X2 was the same as that of X1. The ESI of N2 increased by 83.39%, and N3 had the same ESI as N1. Compared with that of F1, the ESI of F4 increased by 11.48%, F2 had the same ESI as F1, and the ESIs of F3 and F5 decreased by 14.01% and 8.20%, respectively. Therefore, when the winter crops were the same, the sustainability index of the triple-cropping system and the traditional double-cropping system were the same. For winter crop selection, choosing potatoes in the upper reaches of the Yangtze River, either potatoes or forage rape in the middle reaches, and wheat in the lower reaches enhances sustainability.

### 3.7 Sensitivity analysis

The total emergy for the top three contributors was both halved and doubled, which indicated changes in the emergy indices measured. Changes that showed increases or decreases were noted for each emergy index (**Table 7**). Doubling emergy for non-renewable labor across all systems resulted in ESI decreases of 37.9%–40.4% (Sichuan), 33.3%–47.9% (Hubei), 33.3%–35.5% (Hunan), 38.7%–40.7% (Jiangxi), and 43.8%–45.8% (Jiangsu). When emergy was halved, the ESIs increased by 43.5%–50.6% (Sichuan), 81.1%–84.1% (Hubei), 33.1%–37.7% (Hunan), 45.5%–51.6% (Jiangxi), and 63.3%–72.3% (Jiangsu). When emergy was doubled for renewable labor, the ESIs increased by 29.9%–56.9% (Sichuan), 56.9%–80.3% (Hubei), 65.4%–66.4% (Hunan), 28.4%–55.2% (Jiangxi), and 51.3%–69.1% (Jiangsu). When halved, the ESIs decreased by 15.0%–28.6% (Sichuan), 28.6%–40.4% (Hubei), 32.9%–33.4% (Hunan), 14.3%–27.9% (Jiangxi), and 25.8%–34.7% (Jiangsu). Furthermore, fertilizer input was a major contributor to the diversified multi-cropping system. Doubling the emergy for this input decreased ESIs by 18.6%–22.1% (Sichuan), 3.6%–4.2% (Hubei), 23.8%–28.0% (Hunan), 19.7%–22.9% (Jiangxi), and 4.4%–28.3% (Jiangsu). Halving the emergy increased ESIs by 12.9%–16.4% (Sichuan), 1.9%–2.4% (Hubei), 18.5%–24.0% (Hunan), 13.9%–17.4% (Jiangxi), and 1.9%–19.7% (Jiangsu).

**Table 7:** Sensitivity analysis of doubling and halving for emergy inputs.

Test site	Cropping pattern	Emergy input	UEV		UNV		EYR		ELR		ESI	
			Double	Half	Double	Half	Double	Half	Double	Half	Double	Half
Sichuan	R1	Labor–non-renew	50.3%	−25.1%	57.4%	−28.7%	−2.2%	2.3%	57.4%	−28.7%	−37.9%	43.5%
		Fertilizer	23.4%	−11.7%	26.7%	−13.3%	−1.3%	0.9%	26.7%	−13.3%	−22.1%	16.4%
		Labor–renew	6.9%	−3.4%	0.0%	0.0%	−0.4%	0.2%	−35.7%	38.4%	54.8%	−27.6%
	R2	Labor–non-renew	52.7%	−26.4%	60.3%	−30.1%	−2.0%	2.1%	60.3%	−30.1%	−38.8%	46.2%
		Fertilizer	22.1%	−11.0%	25.2%	−12.6%	−1.1%	0.7%	25.2%	−12.6%	−21.0%	15.3%
		Labor–renew	7.2%	−3.6%	0.0%	0.0%	−0.4%	0.2%	−36.5%	40.3%	56.9%	−28.6%
	R3	Labor–non-renew	50.3%	−25.1%	65.0%	−32.5%	−1.6%	1.6%	65.0%	−32.5%	−40.4%	50.6%
		Fertilizer	17.0%	−8.5%	22.0%	−11.0%	−0.7%	0.5%	22.0%	−11.0%	−18.6%	12.9%
		Labor–renew	6.9%	−3.4%	0.0%	0.0%	−0.3%	0.2%	−23.2%	17.8%	29.9%	−15.0%
Hubei	E1	Labor–non-renew	74.2%	−37.1%	85.3%	−42.7%	−2.6%	3.8%	85.3%	−42.7%	−47.4%	81.1%
		Fertilizer	3.7%	−1.8%	4.2%	−2.1%	−0.2%	0.1%	4.2%	−2.1%	−4.2%	2.3%
		Labor–renew	10.1%	−5.1%	0.0%	0.0%	−0.6%	0.3%	−43.8%	63.7%	76.8%	−38.7%
	E2	Labor–non-renew	75.6%	−37.8%	87.0%	−43.5%	−2.2%	3.2%	87.0%	−43.5%	−47.7%	82.7%
		Fertilizer	3.4%	−1.7%	3.9%	−1.9%	−0.2%	0.1%	3.9%	−1.9%	−3.9%	2.1%
		Labor–renew	10.3%	−5.2%	0.0%	0.0%	−0.5%	0.3%	−44.2%	65.6%	78.4%	−39.4%
	E3	Labor–non-renew	76.7%	−38.4%	88.1%	−44.1%	−2.0%	3.0%	88.1%	−44.1%	−47.9%	84.1%
		Fertilizer	3.1%	−1.5%	3.5%	−1.8%	−0.1%	0.1%	3.5%	−1.8%	−3.6%	1.9%
		Labor–renew	10.5%	−5.2%	0.0%	0.0%	−0.4%	0.3%	−44.8%	68.1%	80.3%	−40.4%
	E4	Labor–non-renew	72.7%	−36.3%	87.8%	−43.9%	−1.6%	2.2%	87.8%	−43.9%	−47.6%	82.2%
		Fertilizer	3.7%	−1.9%	4.5%	−2.2%	−0.1%	0.1%	4.5%	−2.2%	−4.4%	2.4%
		Labor–renew	9.9%	−4.9%	0.0%	0.0%	−0.3%	0.2%	−36.5%	40.3%	56.9%	−28.6%
Hunan	X1	Labor–non-renew	42.4%	−21.2%	46.5%	−23.2%	−2.4%	2.2%	46.5%	−23.2%	−33.3%	33.2%
		Fertilizer	31.3%	−15.7%	34.3%	−17.2%	−1.9%	1.5%	34.3%	−17.2%	−27.0%	22.6%
		Labor–renew	5.8%	−2.9%	0.0%	0.0%	−0.4%	0.2%	−40.0%	50.0%	66.0%	−33.2%
	X2	Labor–non-renew	42.8%	−21.4%	46.9%	−23.5%	−2.0%	1.9%	46.9%	−23.5%	−33.3%	33.1%
		Fertilizer	33.4%	−16.7%	36.6%	−18.3%	−1.7%	1.4%	36.6%	−18.3%	−28.0%	24.0%
		Labor–renew	5.8%	−2.9%	0.0%	0.0%	−0.4%	0.2%	−40.1%	50.4%	66.4%	−33.4%
	X3	Labor–non-renew	46.3%	−23.1%	51.2%	−25.6%	−2.5%	2.5%	51.2%	−25.6%	−35.5%	37.7%
		Fertilizer	26.3%	−13.2%	29.1%	−14.6%	−1.6%	1.2%	29.1%	−14.6%	−23.8%	18.5%
		Labor–renew	6.3%	−3.2%	0.0%	0.0%	−0.5%	0.3%	−39.8%	49.4%	65.4%	−32.9%
	X4	Labor–non-renew	46.3%	−23.1%	51.2%	−25.6%	−2.5%	2.5%	51.2%	−25.6%	−35.5%	37.7%
		Fertilizer	26.3%	−13.2%	29.1%	−14.6%	−1.6%	1.2%	29.1%	−14.6%	−23.8%	18.5%
		Labor–renew	6.3%	−3.2%	0.0%	0.0%	−0.5%	0.3%	−39.8%	49.4%	65.4%	−32.9%
Jiangxi	N1	Labor–non-renew	48.8%	−24.4%	56.1%	−28.1%	−4.3%	4.7%	56.1%	−28.1%	−38.7%	45.5%
		Fertilizer	23.0%	−11.5%	26.5%	−13.2%	−2.5%	1.8%	26.5%	−13.2%	−22.9%	17.4%
		Labor–renew	6.7%	−3.3%	0.0%	0.0%	−0.9%	0.5%	−33.8%	34.3%	49.8%	−25.2%
	N2	Labor–non-renew	47.8%	−23.9%	61.8%	−30.9%	−2.3%	2.3%	61.8%	−30.9%	−39.6%	48.1%
		Fertilizer	20.6%	−10.3%	26.7%	−13.3%	−1.2%	0.8%	26.7%	−13.3%	−22.0%	16.3%
		Labor–renew	6.6%	−3.3%	0.0%	0.0%	−0.4%	0.2%	−22.4%	16.9%	28.4%	−14.3%
	N3	Labor–non-renew	54.1%	−27.0%	62.2%	−31.1%	−3.8%	4.4%	62.2%	−31.1%	−40.7%	51.6%
		Fertilizer	19.4%	−9.7%	22.3%	−11.2%	−1.8%	1.2%	22.3%	−11.2%	−19.7%	13.9%
		Labor–renew	7.4%	−3.7%	0.0%	0.0%	−0.8%	0.4%	−36.1%	39.3%	55.2%	−27.9%
Jiangsu	F1	Labor–non-renew	68.0%	−34.0%	80.1%	−40.0%	−2.1%	2.8%	80.1%	−40.0%	−45.6%	71.5%
		Fertilizer	12.0%	−4.9%	14.1%	−5.8%	−0.6%	0.3%	14.1%	−5.8%	−12.8%	6.4%
		Labor–renew	9.3%	−4.6%	0.0%	0.0%	−0.5%	0.3%	−38.1%	44.3%	60.7%	−30.5%
	F2	Labor–non-renew	68.8%	−34.4%	80.9%	−40.5%	−1.9%	2.6%	80.9%	−40.5%	−45.8%	72.3%
		Fertilizer	24.6%	−1.5%	28.9%	−1.8%	−0.9%	0.1%	28.9%	−1.8%	−23.2%	1.9%
		Labor–renew	9.3%	−4.7%	0.0%	0.0%	−0.4%	0.2%	−38.3%	45.0%	61.4%	−30.9%
	F3	Labor–non-renew	68.6%	−34.3%	79.3%	−39.7%	−1.4%	1.9%	79.3%	−39.7%	−45.0%	68.9%
		Fertilizer	3.9%	−8.7%	4.5%	−10.1%	−0.1%	0.3%	4.5%	−10.1%	−4.4%	11.6%
		Labor–renew	9.4%	−4.7%	0.0%	0.0%	−0.3%	0.2%	−41.0%	53.4%	69.1%	−34.7%
	F4	Labor–non-renew	62.8%	−31.4%	75.1%	−37.6%	−2.2%	2.8%	75.1%	−37.6%	−44.2%	64.7%
		Fertilizer	31.3%	−2.5%	37.5%	−3.0%	−1.4%	0.2%	37.5%	−3.0%	−28.3%	3.3%
		Labor–renew	8.5%	−4.3%	0.0%	0.0%	−0.5%	0.3%	−34.2%	35.1%	51.3%	−25.8%
	F5	Labor–non-renew	64.4%	−32.2%	75.1%	−37.5%	−1.6%	2.0%	75.1%	−37.5%	−43.8%	63.3%
		Fertilizer	19.1%	−13.7%	22.2%	−15.9%	−0.7%	0.7%	22.2%	−15.9%	−18.7%	19.7%
		Labor–renew	8.8%	−4.4%	0.0%	0.0%	−0.3%	0.2%	−38.3%	45.0%	61.5%	−30.9%

UEV, unit emergy value; UNV, unit non-renewable value; EYR, emergy yield ratio; ELR, environmental loading ratio; ESI, emergy sustainability index.

## 4 Discussions

### 4.1 Crop productivity and economic benefit

A diversified cropping system enhances resource efficiency, with the size of the benefits depending on crop species combination, temporal and spatial arrangement of crops, and management practices (Cong et al., 2021). This study shows that compared with the traditional double-cropping system, incorporating soybeans into the annual planting system by intercropping with maize has increased the annual productivity and significantly increased the annual economic output (**Figures 3**, **4**). Similar results were also reported by Xia et al. (2023). The wheat–maize/soybean planting system diversifies products with higher outputs, profitability, and temporal yield stability than conventional wheat–maize double cropping. In addition, multi-cropping systems mainly improved the use efficiency of natural resources such as sunlight, temperature, and rain compared to monocropping (Li et al., 2023). Intercropping legumes with maize was a typical partner in terms of biological characteristics, space–time collocation, and resource utilization, which could take full advantage of natural resource access (Zhao et al., 2022).

Previous studies have shown that the introduction of winter crops could produce higher annual crop productivity compared with the winter fallow by making full use of the abundant natural resources (Xian et al., 2023). In this study, the annual productivity and annual economic output of N2 and N3 were significantly higher than those of N1, which was fallow in winter. In addition, the selection of winter crops is very important for the annual productivity of diversified cropping systems (Chen et al., 2018). The annual productivity of R3, E4, N2, X3, and X4 with forage rape and potato as winter crops was significantly higher than other planting patterns in the same area. Similar results could be found in published studies. Garbelini et al. (2022) reported that different crops covered in winter have a significant impact on the annual yield of diversified cropping systems. Xian et al. (2023) reported that in the diversified multi-cropping system, ryegrass or Chinese milk vetch in winter, the former was about four times more productive than the latter. In the present study, compared to the CK, E3, E4, F4, and F5 significantly decreased crop productivity while significantly increasing annual economic output, which highlighted the advantages of the economic value of winter crops in improving the annual economic output. Therefore, the economic value of winter crops should also be considered in the selection of winter crops. Furthermore, compared to the CK, all diversified multi-cropping systems had better economic benefits. It shows that the diversified multi-cropping system could enhance the economic feasibility by introducing high-economic value crops and reducing the fallow period of the cropland.

In general, it could be concluded that a diversified multi-cropping system involving soybeans by intercropping with maize and selecting suitable winter cropping showed great potential for improving crop productivity and economic benefit simultaneously. In this study, the potato–maize/soybean (R3), forage rape–maize/soybean (X3), and potato/maize/soybean (N2 and E4) were recommended as the alternatives to the traditional double-cropping system from the viewpoint of improving the productivity of cultivated land and the annual economic output.

### 4.2 Efficiency of multiple-cropping systems in the Yangtze River Basin

The UEV indicates system production efficiency and relies on the balance between emergy input and output (Wang et al., 2014). This study found that with the same winter crops, the UVEs of triple-cropping systems were higher than the UVE of the CK, indicating that the emergy efficiency was lower, which was similar to the research results of Li et al. (2023). The main reason was that the increased multi-cropping index generally required more resource inputs, but the improvement of soybean yield would possibly still be limited. At the same time, R3, X3, and N2 had decreased UEVs of 48.28%–71.49% compared with CK, which indicated that selecting high-yield winter crops in diversified cropping systems could effectively reduce the UVEs, thereby enhancing the utilization efficiency of input emergy (Li et al., 2023). The utilization ratio of non-renewable resources (UNV) is the ratio of emergy input (NR + PN) to total energy output (Y) of non-renewable resources. The lower the UNV, the higher the utilization efficiency of non-renewable resources. The UNV was defined as the ratio of non-renewable resources emergy input (NR + PN) to the total emergy output. A lower UNV indicates a higher utilization efficiency of non-renewable resources. Namely, a stable planting pattern needs to use less non-renewable resources to reduce the threat to the natural environment (Chen et al., 2021b). The results show that with the same winter crops, the UNVs in triple-cropping systems were higher than the UNV of the CK, indicating that the non-renewable resource utilization efficiency of triple-cropping systems was reduced. The main reason was due to the labor input (RNF = 0.12) brought by the increased crops, which greatly increased the input of non-renewable emergy. Yang et al. (2018) also reported that increased labor input was the primary cause of lower non-renewable resource utilization efficiency in maize–soybean intercropping versus single cropping. R3, X3, and N2 had decreased UNVs of 54.20%–71.80% compared with CK, implying that the efficiency of the diversified multi-cropping system to use non-renewable resources was improved even though the UEVs of the system were reduced. Therefore, from the perspective of system efficiency, potato–spring maize/summer soybean (R3), forage rape–spring maize/summer soybean (X3), and potato/spring maize/summer soybean (N2) were the recommended cropping patterns in the present case because they consumed less emergy and non-renewable resources when generating unit useful output.

### 4.3 Sustainability of multiple-cropping systems in the Yangtze River Basin

The EYR reflects the ability of the cultivation pattern to utilize local resources in external input and quantifies the utilization efficiency of local resources. The higher the EYR value, the greater the dependence of the planting system on natural resources (Eyni-Nargeseh et al., 2023). Compared with that of the double-cropping system, the EYR of the triple-cropping system in the Yangtze River Basin all showed a decline or remained flat. It indicated that increasing the multiple-cropping index improved the agricultural intensification level of cropping systems and reduced the dependence on natural resources (Li et al., 2021). The ELR can be used to measure the pressure on the environmental system under a certain economic situation in a certain region. A higher ELR indicates a higher environmental pressure (Brown and Ulgiati, 2004). Liu et al. (2018b) found that from 1997 to 2016, the ELR of crop production systems in China fluctuated and increased, ranging from 3.39 to 4.59, and the ELR of all planting systems in this study was higher than this level. This may be due to the high level of non-renewable resources such as fertilizers, pesticides, machinery, and fuel oil, which has increased the environmental pressure (Xiao et al., 2022). In this study, compared with the CK, the ELR of the diversified cropping system based on the strip compound planting of maize and soybean decreased or remained flat. In the third mature crop selection, compared with that of maize, the ELR of soybean decreased by 14.32%. The results show that incorporating soybean by intercropping with maize could reduce the environmental pressure, which may be due to the nitrogen fixation of soybean and its interspecific promotion with maize, thus enhancing food production with reduced inputs and environmental impacts. In addition, enhancing biodiversity spatially in the cropping system may promote ecosystem services, such as pest and disease control, carbon sequestration, and soil fertility. These services potentially reduce the dependence on external inputs while maintaining high crop yields and production stability (Zhao et al., 2022). Li et al. (2023a) found that the ELR of the novel triple-cropping system decreased by 5.7% compared with the traditional double-cropping system, which was consistent with our results. ESI is a comprehensive index reflecting the sustainability of the system. An ecosystem with a high EYR and a relatively low ELR was deemed sustainable; otherwise, it was considered unsustainable. In the present case, compared with CK, diversified planting systems that maintain the same winter crops result in a consistent ESI. In the third mature crop selection, compared with maize, the ESI of soybean increased by 18.55%. Switching winter crops to potatoes (R3, E4, and N2), feed rape (X3 and X4), and wheat (F5) led to an ESI increase of 75.27%, 12.05%, and 12.07%, respectively. Li et al. (2023b) showed that the ESI of the triple-cropping system with maize as the main crop was significantly increased compared with the double-cropping system, and the reason was that biogas residue was added into the triple-cropping system to improve fertilizer. The choice of soybean as the third mature crop could reduce the input of non-renewable resources such as fertilizers and pesticides compared with maize. However, the increased labor input made the sustainability of triple-cropping systems the same as that of CK.

A sensitivity analysis was performed to identify the major contributors of emergy in the system. Human labor encompasses both renewable and non-renewable emergy because it is a product of both nature and the economy (Moonilall et al., 2020). Labor (non-renewable) was identified as a top contributor to emergy for all systems. Doubling emergy for non-renewable labor across all systems resulted in a 28.1%–47.9% decrease in the ESI. When the emergy was halved, ESI was increased by 33.1%–84.1%. An inverse trend was observed when emergy was doubled for renewable labor. The ESI increased by 28.4%–80.3% across all diversified multi-cropping systems. When halved, the ESI decreased by 14.3%–40.4%. Furthermore, fertilizer input was a major contributor to the diversified multi-cropping system. Doubling the emergy for this input decreased the ESI of the system by 3.6%–28.3%. Halving the emergy increased ESI by 1.9%–24.0%.

Therefore, from the perspective of reducing environmental burden and increasing environmental sustainability, R3: potato–maize/soybean or E4: potato/maize/soybean (upper reaches of the Yangtze River), N2: forage rape–maize/soybean or X3: potato/maize/soybean (middle reaches of the Yangtze River), and F4: wheat–fresh maize/soybean (lower reaches of the Yangtze River) were recommended.

### 4.4 Implication and strategy

#### 4.4.1 The inclusion of legume crops is a crucial direction of diversified multi-cropping systems

Over recent decades, intensive agriculture in China has successfully realized food security but at the expense of negative environmental impacts. Achieving green transformation of agriculture in China requires a fundamental restructuring of cropping systems (Cong et al., 2021). Incorporating the legumes can enhance crop diversity within an ecosystem, which can increase crop yield and reduce the demand for chemical fertilizers and pesticides due to the combined and interrelated effects of nitrogen (N) provision and non-N effects (suppressed pest and disease and improved soil properties) (George et al., 2022). Factually, the promotion of legumes has served as a strategy for the sustainable development of agriculture in many parts of the world (Xian et al., 2023). In the practice of diversified multi-cropping, the comparison between legumes and other crops has been carried out. For example, Sauvadet et al. (2021) compared the effects of legumes and without legumes on soil fertility in different agroecosystems and showed that legumes improved soil fertility indicators, which was demonstrated through significantly higher plant biomass production in the bioassays (+18%) and soil inorganic N (+26%) compared to the low diversity management. Zhao et al. (2022) synthesized 11,768 yield observations from 462 field experiments and analyzed the impact of legume pre-crops on main crop yield in diversified cropping systems. The results showed that legume-based increased the yield of main crops by 20% compared with non-legume cropping systems. In this study, incorporating the soybean in the multi-cropping system was superior to the traditional double-cropping system in terms of economic benefits and environmental performance. Moreover, in the third mature crop selection, compared with maize, the ELR of soybean decreased by 14.32%. Therefore, the development of the diversified multi-cropping system involving legume crops would be another important direction for the transformation of the agricultural system (Li et al., 2021; Xian et al., 2023).

#### 4.4.2 Winter crop selection plays a crucial role in the efficiency and sustainability of the system

This study shows that multi-cropping could improve economic feasibility and system stability by introducing high-yielding and economically valuable winter crops and reducing the fallow period of arable land. With the development of the economy and society, the shortcomings of the planting system that only contained grain crops were becoming more and more obvious, so China put forward the policy of “grain to fodder, grain to economics” (Li et al., 2023a). Our research shows that potato–maize/soybean (R3), forage rape–maize/soybean (X3), and potato–maize/soybean (N2 and E4) were the best in terms of output value and economic benefit analysis, and the advantages mainly came from the high yield and high economic value of forage rape and potato. The coordinated planting of grain, cash, and feed crops, as well as the integration of livestock and farming systems, has been encouraged in many countries for their better eco-economic benefits (Xian et al., 2023). Oliveira et al. (2024) reported that the integrated crop–livestock and crop–livestock–forestry systems have been widely adopted in the Brazilian Cerrado, which was beneficial in improving soil fertility. Hashmiu et al. (2024) showed that diversification of cash and food crops impacted positively household annual crop income and food security. Higher economic benefit was the most intuitive feeling of farmers, and it was also one of the important favorable factors for the successful promotion of diversified planting systems. Therefore, based on abundant natural resources, feed crops and cash crops should be considered in the selection of winter crops. In developing the advantages of intercropping, it was necessary to fully tap the production potential advantages of winter crops.

#### 4.4.3 Reducing the labor input is the key to improving efficiency and sustainability

This study showed that the triple-cropping system based on maize–soybean strip intercropping exhibited a higher reliance on labor compared to the double-cropping system, which was 22.86%–79.49% higher than the traditional double-cropping system. Previous research reported consistent results as well (Li et al., 2021, 2023b). Compared to other crops, intercropping legumes could reduce the demand for chemical fertilizers. Nevertheless, compared with the double-cropping system, the large increase in the labor force during planting, field maintenance, and harvest reduces the utilization efficiency of non-renewable resources in the planting system. Excessive demand for the labor force may reduce the enthusiasm of farmers for planting crops, especially considering the widespread labor shortages in China (Sun et al., 2017). Bolandnazar et al. (2014) pointed out that replacing labor with more advanced machines would help to improve the efficiency and sustainability of the production system. Wang et al. (2018) suggested that improving labor efficiency and advanced agricultural practices will be effective ways to further improve the economic and environmental sustainability of grain systems in China. Therefore, developing efficient machinery stands as the primary tactic to advance the widespread adoption of the maize–soybean strip compound planting triple-cropping systems.

### 4.5 Limitations

The results show that EYRs between the cropping patterns in the same experimental site almost did not show the difference. The primary reason was that the environmental resources inputted for multi-cropping systems in the same region were the same. At the same time, multi-cropping systems were normally intensive, and high-input farming systems and a large number of purchased materials covered up the influences of environmental resources (Li et al., 2023b). Similar results were also reported by published studies (Guo et al., 2023a; Li et al., 2023b). Therefore, EYR could not well reflect the differences between different multiple-cropping systems in the same experimental site.

Based on field experiments, this study introduced the emergy evaluation method and compared the efficiency and sustainability of multiple-cropping patterns in various regions of the Yangtze River Basin with the double-cropping system as the control. However, at present, the multi-dimensional assessment based on various approaches has been a recent sustained trend in evaluating systems. Basavalingaiah et al. (2022) used both data envelopment analysis and life cycle assessment methodologies to evaluate the socio-economic and environmental sustainability of different production systems. Yang et al. (2019) compared the environmental and economic performance of small and large farms based on emergy evaluation, life cycle assessment methods, and economic analysis. Mohanty et al. (2024) comprehensively evaluated the effects of different cropping systems on inorganic and organic fertilization based on energy and carbon footprint analysis. Therefore, comprehensive evaluation based on a multi-index evaluation framework would be required in the future, and different multiple-cropping systems were compared from a comprehensive point of view.

## 5 Conclusion

In this study, the emergy assessment method was used to quantitatively compare the production efficiency and sustainability of multiple-cropping systems in five representative locations in the Yangtze River Basin. The results show that, compared with the traditional double-cropping system, the diversified multi-cropping system in the Yangtze River valley had lower energy conversion efficiency and utilization efficiency of non-renewable resources, but higher emergy output and annual economic output. Increasing the multiple-cropping index improved the agricultural intensification level of the cropping system, reduced the dependence on natural resources, lightened the environmental burden, and increased the environmental sustainability index. The choice of winter crops plays a vital role in system efficiency and sustainability. Generally speaking, choosing R3: potato–maize/soybean or E4: potato/maize/soybean (upper reaches of the Yangtze River), N2: forage rape–maize/soybean or X3: potato/maize/soybean (middle reaches of the Yangtze River), and F4: wheat–fresh maize/soybean (lower reaches of the Yangtze River) facilitates the coexistence of high economic benefits and environmental sustainability. Furthermore, promoting mechanization development and minimizing labor input are vital strategies for enhancing the efficiency and sustainability of the multiple-cropping systems based on maize–soybean strip intercropping in the Yangtze River Basin.

## Data Availability

The original contributions presented in the study are included in the article/**Supplementary Material**. Further inquiries can be directed to the corresponding author.
