# Supplementary material for: Emergy-based evaluation of production efficiency and sustainability of diversified multi-cropping systems in the Yangtze River Basin
Source: Front Plant Sci. 2024 Nov 8;15:1454130. doi: 10.3389/fpls.2024.1454130 (PMC11581874; doi:10.3389/fpls.2024.1454130)
Supplement: Supplementary file 1 [file Table1.docx]

**Supplementary materials**

Xinhui Lei ^a, b^, Bing Liang ^a, b^, Liang Feng ^a, b^, Xuyang Zhao ^a, b^, Tian Pu ^a, b^, Changbing Yu ^c^, Shushan Wang ^d^, Yafeng Wei ^e^, Shumei Ma ^f^, Xiaochun Wang ^a, b,^ *, Wenyu Yang ^a, b^

^a^ College of Agronomy, Sichuan Agricultural University, Chengdu, 611130, China

^b^ Sichuan Engineering Research Center for Crop Strip Intercropping System, Key Laboratory of Crop Ecophysiology and Farming System in Southwest China (Ministry of Agriculture), Chengdu, 611130, China

^c^ Institute of Oil Crops, Chinese Academy of Agricultural Sciences, Wuhan, 430062, China

^d^ College of Agriculture, Jiangxi Agricultural University, Nanchang, 330045, China

^e^ Jiangsu Institute of Agricultural Sciences along the Yangtze River, Nantong, 226541, China

^f^ Hunan Crop Research Institute, Changsha, 410125, China

Corresponding Author:

* College of Agronomy, Sichuan Agricultural University, 211-Huimin Road, Wenjiang District, Chengdu 611130, PR China. E-mail address: xchwang@sicau.edu.cn (X. Wang).

**Table S1-1.** Emergy analysis of different multi-cropping systems in the Yangtze River Basin (Renshou, Sichuan)

| Items | Unit | RNF | UEV  (sej unit^-1^) | Solar emergy flows (sej ha^-1^ yr^-1^) | | |
| --- | --- | --- | --- | --- | --- | --- |
|  |  |  |  | R1 | R2 | R3 |
| Sunlight | J | 1^a^ | 1.00E+00^a^ | 1.70E+13 | 1.70E+13 | 1.70E+13 |
| Rain chemical energy | J | 1^a^ | 7.00E+03^a^ | 2.25E+14 | 2.25E+14 | 2.25E+14 |
| Wind | J | 1^a^ | 8.00E+02^a^ | 9.76E+11 | 9.76E+11 | 9.76E+11 |
| Renewable natural inputs (R) |  |  |  | 2.25E+14 | 2.25E+14 | 2.25E+14 |
| Topsoil loss | J | 0^a^ | 9.40E+04^a^ | 8.01E+14 | 8.01E+14 | 8.01E+14 |
| Non-renewable natural inputs (NR) |  |  |  | 8.01E+14 | 8.01E+14 | 8.01E+14 |
| N fertilizer | g | 0^b^ | 6.38E+09^b^ | 2.39E+15 | 2.39E+15 | 2.39E+15 |
| P fertilizer | g | 0^b^ | 6.55E+09^b^ | 1.08E+15 | 1.38E+15 | 9.83E+14 |
| K fertilizer | g | 0^b^ | 1.85E+09^b^ | 3.05E+14 | 3.05E+14 | 4.16E+14 |
| Pesticides | g | 0^b^ | 1.89E+10^b^ | 5.29E+14 | 6.05E+14 | 4.91E+14 |
| Plastic film | g | 0^b^ | 2.88E+08^b^ |  | 1.29E+13 | 1.29E+13 |
| Machine and tools | g | 0^a^ | 1.13E+10^c^ | 7.09E+14 | 7.09E+14 | 7.09E+14 |
| Diesel | g | 0^a^ | 6.60E+04^d^ | 2.19E+14 | 2.19E+14 | 2.19E+14 |
| Labor(88%) | h | 0.12^e^ | 6.99E+12^f^ | 8.12E+15 | 9.74E+15 | 1.12E+16 |
| Non-renewable resources purchased (PN) |  |  |  | 1.34E+16 | 1.54E+16 | 1.64E+16 |
| Wheat seed | J | 1^g^ | 9.26E+04^d^ | 6.16E+14 | 6.16E+14 |  |
| Potato tubers | J | 1^g^ | 8.30E+04^h^ |  |  | 3.16E+15 |
| Maize seed | J | 1^g^ | 6.03E+04^d^ | 5.07E+13 | 5.07E+13 | 5.07E+13 |
| Soybean seed | J | 1^g^ | 7.65E+04^d^ |  | 9.18E+13 | 9.18E+13 |
| Labor(12%) | h | 0.12^e^ | 6.99E+12^f^ | 1.11E+15 | 1.33E+15 | 1.53E+15 |
| Purchased renewable resources (PR) |  |  |  | 1.77E+15 | 2.09E+15 | 4.83E+15 |
| Total emergy input (T) |  |  |  | 1.62E+16 | 1.85E+16 | 2.23E+16 |

^a^ UEV and RNF references from (Xu et al. 2019); ^b^ UEV and RNF references from (Chen et al. 2021b); ^c^ UEV references from (Houshyar et al. 2018); ^d^ UEV references from (Jiang et al. 2007); ^e^ RNF references from (Li et al. 2023b); ^f^ UEV references from (Moonilall et al. 2020) ^g^ RNF references from (Wang et al. 2017); ^h^ UEV references from (Li et al. 2023a).

**Table S1-2.** Emergy analysis of different multi-cropping systems in the Yangtze River Basin (Enshi, Hubei)

| Items | Unit | RNF | UEV  (sej unit^-1^) | Solar emergy flows (sej ha^-1^ yr^-1^) | | | |
| --- | --- | --- | --- | --- | --- | --- | --- |
|  |  |  |  | E1 | E2 | E3 | E4 |
| Sunlight | J | 1 | 1.00E+00 | 1.74E+13 | 1.74E+13 | 1.74E+13 | 1.74E+13 |
| Rain chemical energy | J | 1 | 7.00E+03 | 4.67E+14 | 4.67E+14 | 4.67E+14 | 4.67E+14 |
| Wind | J | 1 | 8.00E+02 | 2.11E+12 | 2.11E+12 | 2.11E+12 | 2.11E+12 |
| Renewable natural inputs (R) |  |  |  | 4.67E+14 | 4.67E+14 | 4.67E+14 | 4.67E+14 |
| Topsoil loss | J | 0 | 9.40E+04 | 5.86E+14 | 5.86E+14 | 5.86E+14 | 5.86E+14 |
| Non-renewable natural inputs (NR) |  |  |  | 5.86E+14 | 5.86E+14 | 5.86E+14 | 5.86E+14 |
| N fertilizer | g | 0 | 6.38E+09 | 3.54E+14 | 3.83E+14 | 3.83E+14 | 6.00E+14 |
| P fertilizer | g | 0 | 6.55E+09 | 2.52E+14 | 2.82E+14 | 2.82E+14 | 3.93E+14 |
| K fertilizer | g | 0 | 1.85E+09 | 4.90E+13 | 5.74E+13 | 5.74E+13 | 6.66E+13 |
| Pesticides | g | 0 | 1.89E+10 | 2.27E+14 | 3.02E+14 | 3.02E+14 | 4.16E+14 |
| Machine and tools | g | 0 | 1.13E+10 | 6.12E+14 | 6.12E+14 | 6.12E+14 | 6.12E+14 |
| Diesel | g | 0 | 6.60E+04 | 2.08E+14 | 2.08E+14 | 2.08E+14 | 2.08E+14 |
| Labor(88%) | h | 0.12 | 6.99E+12 | 1.33E+16 | 1.62E+16 | 1.80E+16 | 2.07E+16 |
| Non-renewable resources purchased (PN) |  |  |  | 1.50E+16 | 1.81E+16 | 1.99E+16 | 2.30E+16 |
| Forage rape seed | J | 1^a^ | 8.88E+04 ^b^ | 1.17E+13 | 1.17E+13 |  |  |
| Rape seed | J | 1^a^ | 8.88E+04 ^b^ |  |  | 7.03E+12 |  |
| Potato tubers | J | 1 | 8.30E+04 |  |  |  | 1.52E+15 |
| Maize seed | J | 1 | 6.03E+04 | 3.80E+13 | 3.80E+13 | 3.80E+13 | 3.80E+13 |
| Soybean | J | 1 | 7.65E+04 |  | 6.31E+13 | 6.31E+13 | 6.31E+13 |
| Labor(12%) | J | 1 | 6.99E+12 | 1.81E+15 | 2.21E+15 | 2.46E+15 | 2.82E+15 |
| Purchased renewable resources (PR) | h | 0.12 |  | 1.86E+15 | 2.33E+15 | 2.56E+15 | 4.44E+15 |
| Total emergy input (T) |  |  |  | 1.79E+16 | 2.15E+16 | 2.35E+16 | 2.85E+16 |

^a^ RNF references from (Wang et al. 2017); ^b^ UEV references from (Jiang et al. 2007).

| Items | Unit | RNF | UEV  (sej unit^-1^) | Solar emergy flows (sej ha^-1^ yr^-1^) | | | |
| --- | --- | --- | --- | --- | --- | --- | --- |
|  |  |  |  | X1 | X2 | X3 | X4 |
| Sunlight | J | 1 | 1.00E+00 | 3.12E+13 | 3.12E+13 | 3.12E+13 | 3.12E+13 |
| Rain chemical energy | J | 1 | 7.00E+03 | 4.16E+14 | 4.16E+14 | 4.16E+14 | 4.16E+14 |
| Wind | J | 1 | 8.00E+02 | 6.02E+12 | 6.02E+12 | 6.02E+12 | 6.02E+12 |
| Renewable natural inputs (R) |  |  |  | 4.16E+14 | 4.16E+14 | 4.16E+14 | 4.16E+14 |
| Topsoil loss | J | 0 | 9.40E+04 | 8.90E+14 | 8.90E+14 | 8.90E+14 | 8.90E+14 |
| Non-renewable natural inputs (NR) |  |  |  | 8.90E+14 | 8.90E+14 | 8.90E+14 | 8.90E+14 |
| N fertilizer | g | 0 | 6.38E+09 | 2.47E+15 | 2.90E+15 | 2.11E+15 | 2.11E+15 |
| P fertilizer | g | 0 | 6.55E+09 | 2.65E+15 | 3.61E+15 | 2.28E+15 | 2.28E+15 |
| K fertilizer | g | 0 | 1.85E+09 | 3.33E+14 | 3.95E+14 | 2.29E+14 | 2.29E+14 |
| Pesticides | g | 0 | 1.89E+10 | 3.21E+14 | 3.97E+14 | 3.97E+14 | 3.97E+14 |
| Machine and tools | g | 0 | 1.13E+10 | 1.29E+15 | 1.29E+15 | 1.29E+15 | 1.29E+15 |
| Diesel | g | 0 | 6.60E+04 | 5.48E+14 | 5.48E+14 | 5.48E+14 | 5.48E+14 |
| Labor(88%) | h | 0.12 | 6.99E+12 | 7.38E+15 | 8.86E+15 | 8.12E+15 | 8.12E+15 |
| Non-renewable resources purchased (PN) |  |  |  | 1.50E+16 | 1.80E+16 | 1.50E+16 | 1.50E+16 |
| Rape seed | J | 1 | 8.88E+04 | 3.75E+13 | 3.75E+13 |  |  |
| Forage Rape seed | J | 1 | 8.88E+04 |  |  | 9.38E+12 | 9.38E+12 |
| Maize seed | J | 1 | 6.03E+04 | 5.07E+13 | 5.07E+13 | 5.07E+13 | 5.07E+13 |
| Soybean seed | J | 1 | 7.65E+04 |  | 9.18E+13 | 9.18E+13 | 9.18E+13 |
| Labor(12%) | h | 0.12 | 6.99E+12 | 1.01E+15 | 1.21E+15 | 1.11E+15 | 1.11E+15 |
| Purchased renewable resources (PR) |  |  |  | 1.09E+15 | 1.39E+15 | 1.26E+15 | 1.26E+15 |
| Total emergy input (T) |  |  |  | 1.74E+16 | 2.07E+16 | 1.76E+16 | 1.76E+16 |

**Table S1-3.** Emergy analysis of different multi-cropping systems in the Yangtze River Basin (Xiangyin, Hunan)

**Table S1-4.** Emergy analysis of different multi-cropping systems in the Yangtze River Basin (Jinxian, Jiangxi)

| Items | Unit | RNF | UEV  (sej unit^-1^) | Solar emergy flows (sej ha^-1^ yr^-1^) | | |
| --- | --- | --- | --- | --- | --- | --- |
|  |  |  |  | N1 | N2 | N3 |
| Sunlight | J | 1 | 1.00E+00 | 2.55E+13 | 2.55E+13 | 2.55E+13 |
| Rain chemical energy | J | 1 | 7.00E+03 | 5.90E+14 | 5.90E+14 | 5.90E+14 |
| Wind | J | 1 | 8.00E+02 | 9.92E+12 | 9.92E+12 | 9.92E+12 |
| Renewable natural inputs (R) |  |  |  | 5.90E+14 | 5.90E+14 | 5.90E+14 |
| Topsoil loss | J | 0 | 9.40E+04 | 8.08E+14 | 8.08E+14 | 8.08E+14 |
| Non-renewable natural inputs (NR) |  |  |  | 8.08E+14 | 8.08E+14 | 8.08E+14 |
| N fertilizer | g | 0 | 6.38E+09 | 1.37E+15 | 2.09E+15 | 1.37E+15 |
| P fertilizer | g | 0 | 6.55E+09 | 9.43E+14 | 1.68E+15 | 9.43E+14 |
| K fertilizer | g | 0 | 1.85E+09 | 3.33E+14 | 5.41E+14 | 3.33E+14 |
| Pesticides | g | 0 | 1.89E+10 | 2.55E+14 | 3.78E+14 | 3.50E+14 |
| Machine and tools | g | 0 | 1.13E+10 | 5.14E+14 | 5.14E+14 | 5.14E+14 |
| Diesel | g | 0 | 6.60E+04 | 1.64E+14 | 1.64E+14 | 1.64E+14 |
| Labor(88%) | h | 0.12 | 6.99E+12 | 5.61E+15 | 1.00E+16 | 7.38E+15 |
| Non-renewable resources purchased (PN) |  |  |  | 9.19E+15 | 1.54E+16 | 1.11E+16 |
| Potato tubers | J | 1 | 8.30E+04 |  | 2.63E+15 |  |
| Ryegrass seed | J | 1^a^ | 8.41E+04 ^a^ |  |  | 4.88E+13 |
| Maize seed | J | 1 | 6.03E+04 | 5.07E+13 | 5.07E+13 | 5.07E+13 |
| Soybean seed | J | 1 | 7.65E+04 | 9.18E+13 | 9.18E+13 | 9.18E+13 |
| Labor(12%) | h | 0.12 | 6.99E+12 | 7.65E+14 | 1.37E+15 | 1.01E+15 |
| Purchased renewable resources (PR) |  |  |  | 9.07E+14 | 4.14E+15 | 1.20E+15 |
| Total emergy input (T) |  |  |  | 1.15E+16 | 2.09E+16 | 1.37E+16 |

^a^ UEV and RNF references from (Li et al. 2023b).

**Table S1-5.** Emergy analysis of different multi-cropping systems in the Yangtze River Basin (Rugao, Jiangsu)

| Items | Unit | RNF | UEV  (sej unit^-1^) | Solar emergy flows (sej ha^-1^ yr^-1^) | | | | |
| --- | --- | --- | --- | --- | --- | --- | --- | --- |
|  |  |  |  | F1 | F2 | F3 | F4 | F5 |
| Sunlight | J | 1 | 1.00E+00 | 3.15E+13 | 3.15E+13 | 3.15E+13 | 3.15E+13 | 3.15E+13 |
| Rain chemical energy | J | 1 | 7.00E+03 | 5.12E+14 | 5.12E+14 | 5.12E+14 | 5.12E+14 | 5.12E+14 |
| Wind | J | 1 | 8.00E+02 | 2.74E+12 | 2.74E+12 | 2.74E+12 | 2.74E+12 | 2.74E+12 |
| Renewable natural inputs (R) |  |  |  | 5.12E+14 | 5.12E+14 | 5.12E+14 | 5.12E+14 | 5.12E+14 |
| Topsoil loss | J | 0 | 9.40E+04 | 4.80E+14 | 4.80E+14 | 4.80E+14 | 4.80E+14 | 4.80E+14 |
| Non-renewable natural inputs (NR) |  |  |  | 4.80E+14 | 4.80E+14 | 4.80E+14 | 4.80E+14 | 4.80E+14 |
| N fertilizer | g | 0 | 6.38E+09 | 1.30E+15 | 1.44E+15 | 2.23E+15 | 1.43E+15 | 2.22E+15 |
| P fertilizer | g | 0 | 6.55E+09 | 5.90E+14 | 5.90E+14 | 1.18E+15 | 7.86E+14 | 1.38E+15 |
| K fertilizer | g | 0 | 1.85E+09 | 1.67E+14 | 1.67E+14 | 3.33E+14 | 2.22E+14 | 3.89E+14 |
| Pesticides | g | 0 | 1.89E+10 | 3.40E+14 | 3.78E+14 | 5.29E+14 | 3.21E+14 | 4.73E+14 |
| Machine and tools | g | 0 | 1.13E+10 | 3.22E+14 | 3.22E+14 | 3.22E+14 | 3.22E+14 | 3.22E+14 |
| Diesel | g | 0 | 6.60E+04 | 1.10E+14 | 1.10E+14 | 1.10E+14 | 1.10E+14 | 1.10E+14 |
| Labor(88%) | h | 0.12 | 6.99E+12 | 1.33E+16 | 1.48E+16 | 1.99E+16 | 1.11E+16 | 1.62E+16 |
| Non-renewable resources purchased (PN) |  |  |  | 1.61E+16 | 1.78E+16 | 2.46E+16 | 1.43E+16 | 2.11E+16 |
| faba bean seed | J | 1^a^ | 7.65E+04^b^ | 5.74E+14 | 5.74E+14 | 5.74E+14 |  |  |
| Wheat seed | J | 1 | 9.26E+04 |  |  |  | 7.39E+14 | 7.39E+14 |
| Maize seed | J | 1 | 6.03E+05 | 5.07E+13 | 5.07E+13 | 1.01E+14 | 5.07E+13 | 1.01E+14 |
| Soybean seed | J | 1 | 7.65E+04 |  | 9.18E+13 |  | 9.18E+13 |  |
| Labor(12%) | h | 0.12 | 6.99E+12 | 1.81E+15 | 2.01E+15 | 2.72E+15 | 1.51E+15 | 2.21E+15 |
| Purchased renewable resources (PR) |  |  |  | 2.44E+15 | 2.73E+15 | 3.39E+15 | 2.39E+15 | 3.05E+15 |
| Total emergy input (T) |  |  |  | 1.95E+16 | 2.15E+16 | 2.90E+16 | 1.76E+16 | 2.52E+16 |

^a^ RNF references from (Wang et al. 2017); ^b^ UEV references from (Jiang et al. 2007).

**Table S1-1 Calculation of different planting patterns in the Yangtze River basin (Renshou, Sichuan)**

**R1:**

1. Solar energy:

Land area = 10000 m^2^.

Isolation = 2.13E+09 J/m^2^/yr.

Albedo = 20%.

Energy (J) = 2.13E+09 J/m^2^/yr × (1-20%) ×10000 m^2^ = 1.70E+13 J/ha.

1. Rain chemical energy:

Land area = 10000 m^2^.

Rainfall = 0.650 m/yr.

Conversion = 1000 kg/m^3^.

Gibbs free energy = 4940 J/kg.

Energy (J) = 0.650 m/yr × 10000 m^2^ ×1000 kg/m^3^ ×4940 J/kg = 3.21E+10 J/ha.

1. Wind energy:

Land area = 10000 m^2^.

Wind speed = 1.44 m/s.

Air density = 1.3 kg/m^3^.

Drag coefficient = 0.001.

Time (s) = 3.15E+07 s/yr.

Energy (J) = 10000 m^2^ ×1.3 kg/m^3^ × 0.001 × (1.44)^3^ m/s ×3.15E+7 s = 1.22E+09 J/ha.

1. Net soil loss:

Average soil loss = 18.38 t/ha. (Liu et al. 2023)

Organic matter = 2.05 %.

Organic matter energy = 5400 kcal/kg.

Conversion = 4186 J/kcal.

Energy of net soil loss = 1.838E+04 kg/ha × 0.0205 kg/kg soil × 5400 kcal/kg × 4186 J/kcal = 8.52E+09 J/ha.

1. Nitrogen fertilizer:

N content = 3.75E+05 g/ha.

1. Phosphate fertilizer:

P_2_O_5_ content = 1.65E+05 g/ha.

1. Potash fertilizer:

K_2_O content = 1.65E+05 g/ha.

1. Pesticides:

Quantity = 2.80E+04 g/ha.

1. Mechanical equipment:

Total use = 6.27E+04 g/yr.

1. Diesel:

Total use = 60.0 kg/ha.

Energy content per kg = 5.53E+07 J/kg. (Jiang et al.2007)

Energy (J) = 60.0 kg/ha × 5.53E+07 J/kg = 3.32E+09 J/ha.

1. Labor:

Hours = 1.32E+03 h/ha/yr

1. Seeds:

Wheat：

Quantity = 500.00 kg/ha.

Conversion = 1.33E+07 J/kg.

Energy (J) = 500.00 kg/ha × 1.33E+07 J/kg = 6.65E+09 J/ha.

Corn：

Quantity = 60.00 kg/ha.

Conversion = 1.40E+07 J/kg.

Energy (J) =60.00 kg/ha × 1.40E+07 J/kg = 8.40E+08 J/ha.

**R2:**

1. Solar energy:

Land area = 10000 m^2^.

Isolation = 2.13E+09 J/m^2^/yr.

Albedo = 20%.

Energy (J) = 2.13E+09 J/m^2^/yr × (1-20%) ×10000 m^2^ = 1.70E+13 J/ha.

1. Rain chemical energy:

Land area = 10000 m^2^.

Rainfall = 0.650 m/yr.

Conversion = 1000 kg/m^3^.

Gibbs free energy = 4940 J/kg.

Energy (J) = 0.650 m/yr × 10000 m^2^ ×1000 kg/m^3^ ×4940 J/kg = 3.21E+10 J/ha.

1. Wind energy:

Land area = 10000 m^2^.

Wind speed = 1.44 m/s.

Air density = 1.3 kg/m^3^.

Drag coefficient = 0.001.

Time (s) = 3.15E+07 s/yr.

Energy (J) = 10000 m^2^ ×1.3 kg/m^3^ × 0.001 × (1.44)^3^ m/s ×3.15E+7 s = 1.22E+09 J/ha.

1. Net soil loss:

Average soil loss = 18.38 t/ha. (Liu et al. 2023)

Organic matter = 2.05 %.

Organic matter energy = 5400 kcal/kg.

Conversion = 4186 J/kcal.

Energy of net soil loss = 1.838E+04 kg/ha × 0.0205 kg/kg soil × 5400 kcal/kg × 4186 J/kcal = 8.52E+09 J/ha.

1. Nitrogen fertilizer:

N content = 3.75E+05 g/ha.

1. Phosphate fertilizer:

P_2_O_5_ content = 2.10E+05 g/ha.

1. Potash fertilizer:

K_2_O content = 1.65E+05 g/ha.

1. Pesticides:

Quantity =3.20E+04 g/ha.

1. Plastic film:

Quantity = 4.48E+04 g/ha.

1. Mechanical equipment:

Total use = 6.27E+04 g/yr.

1. Diesel:

Total use = 60.0 kg/ha.

Energy content per kg = 5.53E+07 J/kg. (Jiang et al.2007)

Energy (J) = 60.0 kg/ha × 5.53E+07 J/kg = 3.32E+09 J/ha.

1. Labor:

Hours = 1.58E+03 h/ha/yr

1. Seeds:

Wheat：

Quantity = 500.00 kg/ha.

Conversion = 1.33E+07 J/kg.

Energy (J) = 500.00 kg/ha × 1.33E+07 J/kg = 6.65E+09 J/ha.

Corn：

Quantity = 60.00 kg/ha.

Conversion = 1.40E+07 J/kg.

Energy (J) =60.00 kg/ha × 1.40E+07 J/kg = 8.40E+08 J/ha.

Soybean：

Quantity = 80.00 kg/ha.

Conversion = 1.50E+07 J/kg.

Energy (J) = 80.00 kg/ha ×1.50E+07 J/kg = 1.20E+09 J/ha.

**R3:**

1. Solar energy:

Land area = 10000 m^2^.

Isolation = 2.13E+09 J/m^2^/yr.

Albedo = 20%.

Energy (J) = 2.13E+09 J/m^2^/yr × (1-20%) ×10000 m^2^ = 1.70E+13 J/ha.

1. Rain chemical energy:

Land area = 10000 m^2^.

Rainfall = 0.650 m/yr.

Conversion = 1000 kg/m^3^.

Gibbs free energy = 4940 J/kg.

Energy (J) = 0.650 m/yr × 10000 m^2^ ×1000 kg/m^3^ ×4940 J/kg = 3.21E+10 J/ha.

1. Wind energy:

Land area = 10000 m^2^.

Wind speed = 1.44 m/s.

Air density = 1.3 kg/m^3^.

Drag coefficient = 0.001.

Time (s) = 3.15E+07 s/yr.

Energy (J) = 10000 m^2^ ×1.3 kg/m^3^ × 0.001 × (1.44)^3^ m/s ×3.15E+7 s = 1.22E+09 J/ha.

1. Net soil loss:

Average soil loss = 18.38 t/ha. (Liu et al. 2023)

Organic matter = 2.05 %.

Organic matter energy = 5400 kcal/kg.

Conversion = 4186 J/kcal.

Energy of net soil loss = 1.838E+04 kg/ha × 0.0205 kg/kg soil × 5400 kcal/kg × 4186 J/kcal = 8.52E+09 J/ha.

1. Nitrogen fertilizer:

N content = 3.75E+05 g/ha.

1. Phosphate fertilizer:

P_2_O_5_ content = 1.50E+05 g/ha.

1. Potash fertilizer:

K_2_O content = 2.25E+05 g/ha.

1. Pesticides:

Quantity = 2.60E+04 g/ha.

1. Plastic film:

Quantity = 4.48E+04 g/ha.

1. Mechanical equipment:

Total use = 6.27E+04 g/yr.

1. Diesel:

Total use = 60.0 kg/ha.

Energy content per kg = 5.53E+07 J/kg. (Jiang et al.2007)

Energy (J) = 60.0 kg/ha × 5.53E+07 J/kg = 3.32E+09 J/ha.

1. Labor:

Hours =1.82E+03 h/ha/yr

1. Seeds:

Tubers：

Quantity = 2700.00 kg/ha.

Conversion = 1.41E+07 J/kg.

Energy (J) = 2700.00 kg/ha × 1.41E+07 J/kg = 3.81E+10 J/ha.

Corn：

Quantity = 60.00 kg/ha.

Conversion = 1.40E+07 J/kg.

Energy (J) =60.00 kg/ha × 1.40E+07 J/kg = 8.40E+08 J/ha.

Soybean：

Quantity = 80.00 kg/ha.

Conversion = 1.50E+07 J/kg.

Energy (J) = 80.00 kg/ha ×1.50E+07 J/kg = 1.20E+09 J/ha.

**Table S1-2 Calculation of different planting patterns in the Yangtze River basin (Enshi, Hubei)**

**E1：**

1. Solar energy:

Land area = 10000 m^2^.

Isolation = 2.17E+09 J/m^2^/yr.

Albedo = 20%.

Energy (J) = 2.17E+09 J/m^2^/yr × (1-20%) ×10000 m^2^ = 1.74E+13 J/ha.

1. Rain chemical energy:

Land area = 10000 m^2^.

Rainfall = 1.348 m/yr.

Conversion = 1000 kg/m^3^.

Gibbs free energy = 4940 J/kg.

Energy (J) = 1.348 m/yr × 10000 m^2^ ×1000 kg/m^3^ ×4940 J/kg = 6.67E+10 J/ha.

1. Wind energy:

Land area = 10000 m^2^.

Wind speed = 1.86 m/s.

Air density = 1.3 kg/m^3^.

Drag coefficient = 0.001.

Time (s) = 3.15E+07 s/yr.

Energy (J) = 10000 m^2^ ×1.3 kg/m^3^ × 0.001 × (1.86)^3^ m/s ×3.15E+7 s = 2.64E+09 J/ha.

1. Net soil loss:

Average soil loss = 18.38 t/ha. (Liu et al. 2023)

Organic matter = 1.50 %.

Organic matter energy = 5400 kcal/kg.

Conversion = 4186 J/kcal.

Energy of net soil loss = 1.838E+04 kg/ha × 0.0150 kg/kg soil × 5400 kcal/kg × 4186 J/kcal = 6.23E+09 J/ha.

1. Nitrogen fertilizer:

N content = 5.55E+04 g/ha.

1. Phosphate fertilizer:

P_2_O_5_ content = 3.85E+04 g/ha.

1. Potash fertilizer:

K_2_O content = 2.65E+04 g/ha.

1. Pesticides:

Quantity = 1.20E+04 g/ha.

1. Mechanical equipment:

Total use = 5.42E+04 g/yr.

1. Diesel:

Total use = 57.0 kg/ha.

Energy content per kg = 5.53E+07 J/kg. (Jiang et al.2007)

Energy (J) = 57.0 kg/ha × 5.53E+07 J/kg = 3.15E+09 J/ha.

1. Labor:

Hours =2.16E+03 h/ha/yr

1. Seeds:

Forage rape seed：

Quantity = 5.00 kg/ha.

Conversion = 2.64E+07 J/kg.

Energy (J) = 5.00 kg/ha × 2.64E+07 J/kg = 1.32E+08 J/ha.

Corn：

Quantity = 45.00 kg/ha.

Conversion = 1.40E+07 J/kg.

Energy (J) =45.00 kg/ha × 1.40E+07 J/kg = 6.30E+08 J/ha.

**E2：**

1. Solar energy:

Land area = 10000 m^2^.

Isolation = 2.17E+09 J/m^2^/yr.

Albedo = 20%.

Energy (J) = 2.17E+09 J/m^2^/yr × (1-20%) ×10000 m^2^ = 1.74E+13 J/ha.

1. Rain chemical energy:

Land area = 10000 m^2^.

Rainfall = 1.348 m/yr.

Conversion = 1000 kg/m^3^.

Gibbs free energy = 4940 J/kg.

Energy (J) = 1.348 m/yr × 10000 m^2^ ×1000 kg/m^3^ ×4940 J/kg = 6.67E+10 J/ha.

1. Wind energy:

Land area = 10000 m^2^.

Wind speed = 1.86 m/s.

Air density = 1.3 kg/m^3^.

Drag coefficient = 0.001.

Time (s) = 3.15E+07 s/yr.

Energy (J) = 10000 m^2^ ×1.3 kg/m^3^ × 0.001 × (1.86)^3^ m/s ×3.15E+7 s = 2.64E+09 J/ha.

1. Net soil loss:

Average soil loss = 18.38 t/ha. (Liu et al. 2023)

Organic matter = 1.50 %.

Organic matter energy = 5400 kcal/kg.

Conversion = 4186 J/kcal.

Energy of net soil loss = 1.838E+04 kg/ha × 0.0150 kg/kg soil × 5400 kcal/kg × 4186 J/kcal = 6.23E+09 J/ha.

1. Nitrogen fertilizer:

N content = 6.00E+04 g/ha.

1. Phosphate fertilizer:

P_2_O_5_ content = 4.31E+04 g/ha.

1. Potash fertilizer:

K_2_O content = 3.10E+04 g/ha.

1. Pesticides:

Quantity = 1.60E+04 g/ha.

1. Mechanical equipment:

Total use = 5.42E+04 g/yr.

1. Diesel:

Total use = 57.0 kg/ha.

Energy content per kg = 5.53E+07 J/kg. (Jiang et al.2007)

Energy (J) = 57.0 kg/ha × 5.53E+07 J/kg = 3.15E+09 J/ha.

1. Labor:

Hours =2.64E+03 h/ha/yr

1. Seeds:

Forage rape seed：

Quantity = 5.00 kg/ha.

Conversion = 2.64E+07 J/kg.

Energy (J) = 5.00 kg/ha × 2.64E+07 J/kg = 1.32E+08 J/ha.

Corn：

Quantity = 45.00 kg/ha.

Conversion = 1.40E+07 J/kg.

Energy (J) =45.00 kg/ha × 1.40E+07 J/kg = 6.30E+08 J/ha.

Soybean：

Quantity = 55.00 kg/ha.

Conversion = 1.50E+07 J/kg.

Energy (J) = 55.00 kg/ha ×1.50E+07 J/kg = 8.25E+08 J/ha.

**E3：**

1. Solar energy:

Land area = 10000 m^2^.

Isolation = 2.17E+09 J/m^2^/yr.

Albedo = 20%.

Energy (J) = 2.17E+09 J/m^2^/yr × (1-20%) ×10000 m^2^ = 1.74E+13 J/ha.

1. Rain chemical energy:

Land area = 10000 m^2^.

Rainfall = 1.348 m/yr.

Conversion = 1000 kg/m^3^.

Gibbs free energy = 4940 J/kg.

Energy (J) = 1.348 m/yr × 10000 m^2^ ×1000 kg/m^3^ ×4940 J/kg = 6.67E+10 J/ha.

1. Wind energy:

Land area = 10000 m^2^.

Wind speed = 1.86 m/s.

Air density = 1.3 kg/m^3^.

Drag coefficient = 0.001.

Time (s) = 3.15E+07 s/yr.

Energy (J) = 10000 m^2^ ×1.3 kg/m^3^ × 0.001 × (1.86)^3^ m/s ×3.15E+7 s = 2.64E+09 J/ha.

1. Net soil loss:

Average soil loss = 18.38 t/ha. (Liu et al. 2023)

Organic matter = 1.50 %.

Organic matter energy = 5400 kcal/kg.

Conversion = 4186 J/kcal.

Energy of net soil loss = 1.838E+04 kg/ha × 0.0150 kg/kg soil × 5400 kcal/kg × 4186 J/kcal = 6.23E+09 J/ha.

1. Nitrogen fertilizer:

N content = 6.00E+04 g/ha.

1. Phosphate fertilizer:

P_2_O_5_ content = 4.31E+04 g/ha.

1. Potash fertilizer:

K_2_O content = 3.10E+04 g/ha.

1. Pesticides:

Quantity = 1.60E+04 g/ha.

1. Mechanical equipment:

Total use = 5.42E+04 g/yr.

1. Diesel:

Total use = 57.0 kg/ha.

Energy content per kg = 5.53E+07 J/kg. (Jiang et al.2007)

Energy (J) = 57.0 kg/ha × 5.53E+07 J/kg = 3.15E+09 J/ha.

1. Labor:

Hours =2.93E+03 h/ha/yr

1. Seeds:

Rape seed：

Quantity = 3.00 kg/ha.

Conversion = 2.64E+07 J/kg.

Energy (J) = 3.0 kg/ha × 2.64E+07 J/kg = 7.92E+07 J/ha.

Corn：

Quantity = 45.00 kg/ha.

Conversion = 1.40E+07 J/kg.

Energy (J) =45.00 kg/ha × 1.40E+07 J/kg = 6.30E+08 J/ha.

Soybean：

Quantity = 55.00 kg/ha.

Conversion = 1.50E+07 J/kg.

Energy (J) = 55.00 kg/ha ×1.50E+07 J/kg = 8.25E+08 J/ha.

**E4：**

1. Solar energy:

Land area = 10000 m^2^.

Isolation = 2.17E+09 J/m^2^/yr.

Albedo = 20%.

Energy (J) = 2.17E+09 J/m^2^/yr × (1-20%) ×10000 m^2^ = 1.74E+13 J/ha.

1. Rain chemical energy:

Land area = 10000 m^2^.

Rainfall = 1.348 m/yr.

Conversion = 1000 kg/m^3^.

Gibbs free energy = 4940 J/kg.

Energy (J) = 1.348 m/yr × 10000 m^2^ ×1000 kg/m^3^ ×4940 J/kg = 6.67E+10 J/ha.

1. Wind energy:

Land area = 10000 m^2^.

Wind speed = 1.86 m/s.

Air density = 1.3 kg/m^3^.

Drag coefficient = 0.001.

Time (s) = 3.15E+07 s/yr.

Energy (J) = 10000 m^2^ ×1.3 kg/m^3^ × 0.001 × (1.86)^3^ m/s ×3.15E+7 s = 2.64E+09 J/ha.

1. Net soil loss:

Average soil loss = 18.38 t/ha. (Liu et al. 2023)

Organic matter = 1.50 %.

Organic matter energy = 5400 kcal/kg.

Conversion = 4186 J/kcal.

Energy of net soil loss = 1.838E+04 kg/ha × 0.0150 kg/kg soil × 5400 kcal/kg × 4186 J/kcal = 6.23E+09 J/ha.

1. Nitrogen fertilizer:

N content = 9.40E+04 g/ha.

1. Phosphate fertilizer:

P_2_O_5_ content = 6.00E+04 g/ha.

1. Potash fertilizer:

K_2_O content = 3.60E+04 g/ha.

1. Pesticides:

Quantity = 2.20E+04 g/ha.

1. Mechanical equipment:

Total use = 5.42E+04 g/yr.

1. Diesel:

Total use = 57.0 kg/ha.

Energy content per kg = 5.53E+07 J/kg. (Jiang et al.2007)

Energy (J) = 57.0 kg/ha × 5.53E+07 J/kg = 3.15E+09 J/ha.

1. Labor:

Hours =3.36E+03 h/ha/yr

1. Seeds:

Tubers：

Quantity = 1300.0 kg/ha.

Conversion = 1.41E+07 J/kg.

Energy (J) = 1300.0 kg/ha × 1.41E+07 J/kg = 1.83E+10 J/ha.

Corn：

Quantity = 45.00 kg/ha.

Conversion = 1.40E+07 J/kg.

Energy (J) =45.00 kg/ha × 1.40E+07 J/kg = 6.30E+08 J/ha.

Soybean：

Quantity = 55.00 kg/ha.

Conversion = 1.50E+07 J/kg.

Energy (J) = 55.00 kg/ha ×1.50E+07 J/kg = 8.25E+08 J/ha.

**Table S1-3 Calculation of different planting patterns in the Yangtze River basin (Xiangyin, Hunan)**

**X1:**

1. Solar energy:

Land area = 10000 m^2^.

Isolation = 3.90E+09 J/m^2^/yr.

Albedo = 20%.

Energy (J) = 3.90E+09 J/m^2^/yr × (1-20%) ×10000 m^2^ = 3.12E+13 J/ha.

1. Rain chemical energy:

Land area = 10000 m^2^.

Rainfall = 1.200 m/yr.

Conversion = 1000 kg/m^3^.

Gibbs free energy = 4940 J/kg.

Energy (J) = 1.200 m/yr × 10000 m^2^ ×1000 kg/m^3^ ×4940 J/kg = 5.94E+10 J/ha.

1. Wind energy:

Land area = 10000 m^2^.

Wind speed = 2.64 m/s.

Air density = 1.3 kg/m^3^.

Drag coefficient = 0.001.

Time (s) = 3.15E+07 s/yr.

Energy (J) = 10000 m^2^ ×1.3 kg/m^3^ × 0.001 × (2.64)^3^ m/s ×3.15E+7 s = 7.53E+09 J/ha.

1. Net soil loss:

Average soil loss = 18.38 t/ha. (Liu et al.2023)

Organic matter = 2.28 %.

Organic matter energy = 5400 kcal/kg.

Conversion = 4186 J/kcal.

Energy of net soil loss = 1.838E+04 kg/ha × 0.0228 kg/kg soil × 5400 kcal/kg × 4186 J/kcal = 9.47E+09 J/ha.

1. Nitrogen fertilizer:

N content = 3.87E+05 g/ha.

1. Phosphate fertilizer:

P_2_O_5_ content =4.05E+05 g/ha.

1. Potash fertilizer:

K_2_O content = 1.80E+05 g/ha.

1. Pesticides:

Quantity = 1.70E+04g/ha.

1. Mechanical equipment:

Total use = 1.14E+05 g/yr.

1. Diesel:

Total use = 150.0 kg/ha.

Energy content per kg = 5.53E+07 J/kg. (Jiang et al.2007)

Energy (J) = 150.0 kg/ha × 5.53E+07 J/kg = 8.30E+09 J/ha.

1. Labor:

Hours =1.20E+03 h/ha/yr

1. Seeds:

Rape seed：

Quantity = 16.00 kg/ha.

Conversion = 2.64E+07 J/kg.

Energy (J) = 16.00 kg/ha × 2.64E+07 J/kg = 4.22E+08 J/ha.

Corn：

Quantity = 60.00 kg/ha.

Conversion = 1.40E+07 J/kg.

Energy (J) =60.00 kg/ha × 1.40E+07 J/kg = 8.40E+08 J/ha.

**X2:**

1. Solar energy:

Land area = 10000 m^2^.

Isolation = 3.90E+09 J/m^2^/yr.

Albedo = 20%.

Energy (J) = 3.90E+09 J/m^2^/yr × (1-20%) ×10000 m^2^ = 3.12E+13 J/ha.

1. Rain chemical energy:

Land area = 10000 m^2^.

Rainfall = 1.200 m/yr.

Conversion = 1000 kg/m^3^.

Gibbs free energy = 4940 J/kg.

Energy (J) = 1.200 m/yr × 10000 m^2^ ×1000 kg/m^3^ ×4940 J/kg = 5.94E+10 J/ha.

1. Wind energy:

Land area = 10000 m^2^.

Wind speed = 2.64 m/s.

Air density = 1.3 kg/m^3^.

Drag coefficient = 0.001.

Time (s) = 3.15E+07 s/yr.

Energy (J) = 10000 m^2^ ×1.3 kg/m^3^ × 0.001 × (2.64)^3^ m/s ×3.15E+7 s = 7.53E+09 J/ha.

1. Net soil loss:

Average soil loss = 18.38 t/ha. (Liu et al.2023)

Organic matter = 2.28 %.

Organic matter energy = 5400 kcal/kg.

Conversion = 4186 J/kcal.

Energy of net soil loss = 1.838E+04 kg/ha × 0.0228 kg/kg soil × 5400 kcal/kg × 4186 J/kcal = 9.47E+09 J/ha.

1. Nitrogen fertilizer:

N content = 4.55E+05 g/ha.

1. Phosphate fertilizer:

P_2_O_5_ content = 5.51E+05 g/ha.

1. Potash fertilizer:

K_2_O content = 2.14E+05 g/ha.

1. Pesticides:

Quantity = 2.10E+04 g/ha.

1. Mechanical equipment:

Total use = 1.14E+05 g/yr.

1. Diesel:

Total use = 150.0 kg/ha.

Energy content per kg = 5.53E+07 J/kg. (Jiang et al.2007)

Energy (J) = 150.0 kg/ha × 5.53E+07 J/kg = 8.30E+09 J/ha.

1. Labor:

Hours =1.44E+03 h/ha/yr

1. Seeds:

Rape seed：

Quantity = 16.0 kg/ha.

Conversion = 2.64E+07 J/kg.

Energy (J) = 16.0kg/ha × 2.64E+07 J/kg = 4.22E+08 J/ha.

Corn：

Quantity = 60.00 kg/ha.

Conversion = 1.40E+07 J/kg.

Energy (J) =60.00 kg/ha × 1.40E+07 J/kg = 8.40E+08 J/ha.

Soybean：

Quantity = 80.00 kg/ha.

Conversion = 1.50E+07 J/kg.

Energy (J) = 80.00 kg/ha ×1.50E+07 J/kg = 1.20E+09 J/ha.

**X3:**

1. Solar energy:

Land area = 10000 m^2^.

Isolation = 3.90E+09 J/m^2^/yr.

Albedo = 20%.

Energy (J) = 3.90E+09 J/m^2^/yr × (1-20%) ×10000 m^2^ = 3.12E+13 J/ha.

1. Rain chemical energy:

Land area = 10000 m^2^.

Rainfall = 1.200 m/yr.

Conversion = 1000 kg/m^3^.

Gibbs free energy = 4940 J/kg.

Energy (J) = 1.200 m/yr × 10000 m^2^ ×1000 kg/m^3^ ×4940 J/kg = 5.94E+10 J/ha.

1. Wind energy:

Land area = 10000 m^2^.

Wind speed = 2.64 m/s.

Air density = 1.3 kg/m^3^.

Drag coefficient = 0.001.

Time (s) = 3.15E+07 s/yr.

Energy (J) = 10000 m^2^ ×1.3 kg/m^3^ × 0.001 × (2.64)^3^ m/s ×3.15E+7 s = 7.53E+09 J/ha.

1. Net soil loss:

Average soil loss = 18.38 t/ha. (Liu et al.2023)

Organic matter = 2.28 %.

Organic matter energy = 5400 kcal/kg.

Conversion = 4186 J/kcal.

Energy of net soil loss = 1.838E+04 kg/ha × 0.0228 kg/kg soil × 5400 kcal/kg × 4186 J/kcal = 9.47E+09 J/ha.

1. Nitrogen fertilizer:

N content = 3.31E+05 g/ha.

1. Phosphate fertilizer:

P_2_O_5_ content = 3.48E+05 g/ha.

1. Potash fertilizer:

K_2_O content = 1.24E+05 g/ha.

1. Pesticides:

Quantity = 2.10E+04 g/ha.

1. Mechanical equipment:

Total use = 1.14E+05 g/yr.

1. Diesel:

Total use = 150.0 kg/ha.

Energy content per kg = 5.53E+07 J/kg. (Jiang et al.2007)

Energy (J) = 150.0 kg/ha × 5.53E+07 J/kg = 8.30E+09 J/ha.

1. Labor:

Hours =1.32E+03 h/ha/yr

1. Seeds:

Forage rape seed：

Quantity = 4.00 kg/ha.

Conversion = 2.64E+07 J/kg.

Energy (J) = 4.00 kg/ha × 2.64E+07 J/kg = 1.06E+08 J/ha.

Corn：

Quantity = 60.00 kg/ha.

Conversion = 1.40E+07 J/kg.

Energy (J) =60.00 kg/ha × 1.40E+07 J/kg = 8.40E+08 J/ha.

Soybean：

Quantity = 80.00 kg/ha.

Conversion = 1.50E+07 J/kg.

Energy (J) = 80.00 kg/ha ×1.50E+07 J/kg = 1.20E+09 J/ha.

**X4:**

1. Solar energy:

Land area = 10000 m^2^.

Isolation = 3.90E+09 J/m^2^/yr.

Albedo = 20%.

Energy (J) = 3.90E+09 J/m^2^/yr × (1-20%) ×10000 m^2^ = 3.12E+13 J/ha.

1. Rain chemical energy:

Land area = 10000 m^2^.

Rainfall = 1.200 m/yr.

Conversion = 1000 kg/m^3^.

Gibbs free energy = 4940 J/kg.

Energy (J) = 1.200 m/yr × 10000 m^2^ ×1000 kg/m^3^ ×4940 J/kg = 5.94E+10 J/ha.

1. Wind energy:

Land area = 10000 m^2^.

Wind speed = 2.64 m/s.

Air density = 1.3 kg/m^3^.

Drag coefficient = 0.001.

Time (s) = 3.15E+07 s/yr.

Energy (J) = 10000 m^2^ ×1.3 kg/m^3^ × 0.001 × (2.64)^3^ m/s ×3.15E+7 s = 7.53E+09 J/ha.

1. Net soil loss:

Average soil loss = 18.38 t/ha. (Liu et al.2023)

Organic matter = 2.28 %.

Organic matter energy = 5400 kcal/kg.

Conversion = 4186 J/kcal.

Energy of net soil loss = 1.838E+04 kg/ha × 0.0228 kg/kg soil × 5400 kcal/kg × 4186 J/kcal = 9.47E+09 J/ha.

1. Nitrogen fertilizer:

N content = 3.31E+05 g/ha.

1. Phosphate fertilizer:

P_2_O_5_ content = 3.48E+05 g/ha.

1. Potash fertilizer:

K_2_O content = 1.24E+05 g/ha.

1. Pesticides:

Quantity = 2.10E+04 g/ha.

1. Mechanical equipment:

Total use = 1.14E+05 g/yr.

1. Diesel:

Total use = 150.0 kg/ha.

Energy content per kg = 5.53E+07 J/kg. (Jiang et al.2007)

Energy (J) = 150.0 kg/ha × 5.53E+07 J/kg = 8.30E+09 J/ha.

1. Labor:

Hours =1.32E+03 h/ha/yr

1. Seeds:

Forage rape seed：

Quantity = 4.00 kg/ha.

Conversion = 2.64E+07 J/kg.

Energy (J) = 4.00 kg/ha × 2.64E+07 J/kg = 1.06E+08 J/ha.

Corn：

Quantity = 60.00 kg/ha.

Conversion = 1.40E+07 J/kg.

Energy (J) =60.00 kg/ha × 1.40E+07 J/kg = 8.40E+08 J/ha.

Soybean：

Quantity = 80.00 kg/ha.

Conversion = 1.50E+07 J/kg.

Energy (J) = 80.00 kg/ha ×1.50E+07 J/kg = 1.20E+09 J/ha.

**Table S1-4 Calculation of different planting patterns in the Yangtze River basin (Jinxian, Jiangxi)**

**N1:**

1. Solar energy:

Land area = 10000 m^2^.

Isolation = 3.18E+09 J/m^2^/yr.

Albedo = 20%.

Energy (J) = 3.18E+09 J/m^2^/yr × (1-20%) ×10000 m^2^ = 2.55E+13 J/ha.

1. Rain chemical energy:

Land area = 10000 m^2^.

Rainfall = 1.705 m/yr.

Conversion = 1000 kg/m^3^.

Gibbs free energy = 4940 J/kg.

Energy (J) = 1.705 m/yr × 10000 m^2^ ×1000 kg/m^3^ ×4940 J/kg = 8.43E+10 J/ha.

1. Wind energy:

Land area = 10000 m^2^.

Wind speed = 3.12 m/s.

Air density = 1.3 kg/m^3^.

Drag coefficient = 0.001.

Time (s) = 3.15E+07 s/yr.

Energy (J) = 10000 m^2^ ×1.3 kg/m^3^ × 0.001 × (3.12)^3^ m/s ×3.15E+7 s = 1.24E+10 J/ha.

1. Net soil loss:

Average soil loss = 18.38 t/ha. (Liu et al. 2023)

Organic matter = 2.07 %.

Organic matter energy = 5400 kcal/kg.

Conversion = 4186 J/kcal.

Energy of net soil loss = 1.838E+04 kg/ha × 0.0207 kg/kg soil × 5400 kcal/kg × 4186 J/kcal = 8.60E+09 J/ha.

1. Nitrogen fertilizer:

N content = 2.15E+05 g/ha.

1. Phosphate fertilizer:

P_2_O_5_ content = 1.44E+05 g/ha.

1. Potash fertilizer:

K_2_O content = 1.80E+05 g/ha.

1. Pesticides:

Quantity = 1.35E+04 g/ha.

1. Mechanical equipment:

Total use = 4.55E+04 g/yr.

1. Diesel:

Total use = 45.0 kg/ha.

Energy content per kg = 5.53E+07 J/kg. (Jiang et al.2007)

Energy (J) = 45.0 kg/ha × 5.53E+07 J/kg = 2.49E+09 J/ha.

1. Labor:

Hours =9.12E+02 h/ha/yr

1. Seeds:

Corn：

Quantity = 60.00 kg/ha.

Conversion = 1.40E+07 J/kg.

Energy (J) =60.00 kg/ha × 1.40E+07 J/kg = 8.40E+08 J/ha.

Soybean：

Quantity = 80.00 kg/ha.

Conversion = 1.50E+07 J/kg.

Energy (J) = 80.00 kg/ha ×1.50E+07 J/kg = 1.20E+09 J/ha.

**N2:**

1. Solar energy:

Land area = 10000 m^2^.

Isolation = 3.18E+09 J/m^2^/yr.

Albedo = 20%.

Energy (J) = 3.18E+09 J/m^2^/yr × (1-20%) ×10000 m^2^ = 2.55E+13 J/ha.

1. Rain chemical energy:

Land area = 10000 m^2^.

Rainfall = 1.705 m/yr.

Conversion = 1000 kg/m^3^.

Gibbs free energy = 4940 J/kg.

Energy (J) = 1.705 m/yr × 10000 m^2^ ×1000 kg/m^3^ ×4940 J/kg = 8.43E+10 J/ha.

1. Wind energy:

Land area = 10000 m^2^.

Wind speed = 3.12 m/s.

Air density = 1.3 kg/m^3^.

Drag coefficient = 0.001.

Time (s) = 3.15E+07 s/yr.

Energy (J) = 10000 m^2^ ×1.3 kg/m^3^ × 0.001 × (3.12)^3^ m/s ×3.15E+7 s = 1.24E+10 J/ha.

1. Net soil loss:

Average soil loss = 18.38 t/ha. (Liu et al. 2023)

Organic matter = 2.07 %.

Organic matter energy = 5400 kcal/kg.

Conversion = 4186 J/kcal.

Energy of net soil loss = 1.838E+04 kg/ha × 0.0207 kg/kg soil × 5400 kcal/kg × 4186 J/kcal = 8.60E+09 J/ha.

1. Nitrogen fertilizer:

N content = 3.28E+05 g/ha.

1. Phosphate fertilizer:

P_2_O_5_ content = 2.56E+05 g/ha.

1. Potash fertilizer:

K_2_O content = 2.92E+05 g/ha.

1. Pesticides:

Quantity = 2.00E+04 g/ha.

1. Mechanical equipment:

Total use = 4.55E+04 g/yr.

1. Diesel:

Total use = 45.0 kg/ha.

Energy content per kg = 5.53E+07 J/kg. (Jiang et al.2007)

Energy (J) = 45.0 kg/ha × 5.53E+07 J/kg = 2.49E+09 J/ha.

1. Labor:

Hours =1.63E+03 h/ha/yr

1. Seeds:

Tubers：

Quantity = 2250.00 kg/ha.

Conversion = 1.41E+07 J/kg.

Energy (J) = 2250.00 kg/ha × 1.41E+07 J/kg = 3.17E+10 J/ha.

Corn：

Quantity = 60.00 kg/ha.

Conversion = 1.40E+07 J/kg.

Energy (J) =60.00 kg/ha × 1.40E+07 J/kg = 8.40E+08 J/ha.

Soybean：

Quantity = 80.00 kg/ha.

Conversion = 1.50E+07 J/kg.

Energy (J) = 80.00 kg/ha ×1.50E+07 J/kg = 1.20E+09 J/ha.

**N3:**

1. Solar energy:

Land area = 10000 m^2^.

Isolation = 3.18E+09 J/m^2^/yr.

Albedo = 20%.

Energy (J) = 3.18E+09 J/m^2^/yr × (1-20%) ×10000 m^2^ = 2.55E+13 J/ha.

1. Rain chemical energy:

Land area = 10000 m^2^.

Rainfall = 1.705 m/yr.

Conversion = 1000 kg/m^3^.

Gibbs free energy = 4940 J/kg.

Energy (J) = 1.705 m/yr × 10000 m^2^ ×1000 kg/m^3^ ×4940 J/kg = 8.43E+10 J/ha.

1. Wind energy:

Land area = 10000 m^2^.

Wind speed = 3.12 m/s.

Air density = 1.3 kg/m^3^.

Drag coefficient = 0.001.

Time (s) = 3.15E+07 s/yr.

Energy (J) = 10000 m^2^ ×1.3 kg/m^3^ × 0.001 × (3.12)^3^ m/s ×3.15E+7 s = 1.24E+10 J/ha.

1. Net soil loss:

Average soil loss = 18.38 t/ha. (Liu et al. 2023)

Organic matter = 2.07 %.

Organic matter energy = 5400 kcal/kg.

Conversion = 4186 J/kcal.

Energy of net soil loss = 1.838E+04 kg/ha × 0.0207 kg/kg soil × 5400 kcal/kg × 4186 J/kcal = 8.60E+09 J/ha.

1. Nitrogen fertilizer:

N content = 2.15E+05 g/ha.

1. Phosphate fertilizer:

P_2_O_5_ content = 1.44E+05 g/ha.

1. Potash fertilizer:

K_2_O content =1.80E+05 g/ha.

1. Pesticides:

Quantity = 1.85E+04 g/ha.

1. Mechanical equipment:

Total use = 4.55E+04 g/yr.

1. Diesel:

Total use = 45.0 kg/ha.

Energy content per kg = 5.53E+07 J/kg. (Jiang et al.2007)

Energy (J) = 45.0 kg/ha × 5.53E+07 J/kg = 2.49E+09 J/ha.

1. Labor:

Hours =1.20E+03 h/ha/yr

1. Seeds:

Ryegrass：

Quantity = 40.00 kg/ha.

Conversion = 1.45E+07 J/kg.

Energy (J) = 40.00 kg/ha × 1.45E+07 J/kg = 5.80E+08 J/ha.

Corn：

Quantity = 60.00 kg/ha.

Conversion = 1.40E+07 J/kg.

Energy (J) =60.00 kg/ha × 1.40E+07 J/kg = 8.40E+08 J/ha.

Soybean：

Quantity = 80.00 kg/ha.

Conversion = 1.50E+07 J/kg.

Energy (J) = 80.00 kg/ha ×1.50E+07 J/kg = 1.20E+09 J/ha.

**Table S1-5 Calculation of different planting patterns in the Yangtze River basin (Rugao, Jiangsu)**

**F1:**

1. Solar energy:

Land area = 10000 m^2^.

Isolation = 3.94E+09 J/m^2^/yr.

Albedo = 20%.

Energy (J) = 3.94E+09 J/m^2^/yr × (1-20%) ×10000 m^2^ = 3.15E+13 J/ha.

1. Rain chemical energy:

Land area = 10000 m^2^.

Rainfall = 1.478 m/yr.

Conversion = 1000 kg/m^3^.

Gibbs free energy = 4940 J/kg.

Energy (J) = 1.478 m/yr × 10000 m^2^ ×1000 kg/m^3^ ×4940 J/kg = 7.31E+10 J/ha.

1. Wind energy:

Land area = 10000 m^2^.

Wind speed = 2.03 m/s.

Air density = 1.3 kg/m^3^.

Drag coefficient = 0.001.

Time (s) = 3.15E+07 s/yr.

Energy (J) = 10000 m^2^ ×1.3 kg/m^3^ × 0.001 × (2.03)^3^ m/s ×3.15E+7 s = 3.43E+09 J/ha.

1. Net soil loss:

Average soil loss = 18.38 t/ha. (Liu et al. 2023)

Organic matter = 1.23 %.

Organic matter energy = 5400 kcal/kg.

Conversion = 4186 J/kcal.

Energy of net soil loss = 1.838E+04 kg/ha × 0.0123 kg/kg soil × 5400 kcal/kg × 4186 J/kcal = 5.11E+09 J/ha.

1. Nitrogen fertilizer:

N content = 2.03E+05 g/ha.

1. Phosphate fertilizer:

P_2_O_5_ content =9.00E+04 g/ha.

1. Potash fertilizer:

K_2_O content =9.00E+04 g/ha.

1. Pesticides:

Quantity = 1.80E+04 g/ha.

1. Mechanical equipment:

Total use = 2.85E+04 g/yr.

1. Diesel:

Total use = 30.0 kg/ha.

Energy content per kg = 5.53E+07 J/kg. (Jiang et al.2007)

Energy (J) = 30.0 kg/ha × 5.53E+07 J/kg = 1.66E+09 J/ha.

1. Labor:

Hours =2.16E+03 h/ha/yr

1. Seeds:

Corn：

Quantity = 60.00 kg/ha.

Conversion = 1.40E+07 J/kg.

Energy (J) =60.00 kg/ha × 1.40E+07 J/kg = 8.40E+08 J/ha.

broad bean：

Quantity = 500.00 kg/ha.

Conversion = 1.50E+07 J/kg.

Energy (J) = 500.00 kg/ha ×1.50E+07 J/kg = 7.50E+09 J/ha.

**F2:**

1. Solar energy:

Land area = 10000 m^2^.

Isolation = 3.94E+09 J/m^2^/yr.

Albedo = 20%.

Energy (J) = 3.94E+09 J/m^2^/yr × (1-20%) ×10000 m^2^ = 3.15E+13 J/ha.

1. Rain chemical energy:

Land area = 10000 m^2^.

Rainfall = 1.478 m/yr.

Conversion = 1000 kg/m^3^.

Gibbs free energy = 4940 J/kg.

Energy (J) = 1.478 m/yr × 10000 m^2^ ×1000 kg/m^3^ ×4940 J/kg = 7.31E+10 J/ha.

1. Wind energy:

Land area = 10000 m^2^.

Wind speed = 2.03 m/s.

Air density = 1.3 kg/m^3^.

Drag coefficient = 0.001.

Time (s) = 3.15E+07 s/yr.

Energy (J) = 10000 m^2^ ×1.3 kg/m^3^ × 0.001 × (2.03)^3^ m/s ×3.15E+7 s = 3.43E+09 J/ha.

1. Net soil loss:

Average soil loss = 18.38 t/ha. (Liu et al. 2023)

Organic matter = 1.23 %.

Organic matter energy = 5400 kcal/kg.

Conversion = 4186 J/kcal.

Energy of net soil loss = 1.838E+04 kg/ha × 0.0123 kg/kg soil × 5400 kcal/kg × 4186 J/kcal = 5.11E+09 J/ha.

1. Nitrogen fertilizer:

N content =2.26E+05 g/ha.

1. Phosphate fertilizer:

P_2_O_5_ content =9.00E+04 g/ha.

1. Potash fertilizer:

K_2_O content = 9.00E+04 g/ha.

1. Pesticides:

Quantity = 2.00E+04 g/ha.

1. Mechanical equipment:

Total use = 2.85E+04 g/yr.

1. Diesel:

Total use = 30.0 kg/ha.

Energy content per kg = 5.53E+07 J/kg. (Jiang et al.2007)

Energy (J) = 30.0 kg/ha × 5.53E+07 J/kg = 1.66E+09 J/ha.

1. Labor:

Hours =2.40E+03 h/ha/yr

1. Seeds:

Corn：

Quantity = 60.00 kg/ha.

Conversion = 1.40E+07 J/kg.

Energy (J) =60.00 kg/ha × 1.40E+07 J/kg = 8.40E+08 J/ha.

Soybean：

Quantity = 80.00 kg/ha.

Conversion = 1.50E+07 J/kg.

Energy (J) = 80.00 kg/ha ×1.50E+07 J/kg = 1.20E+09 J/ha.

broad bean：

Quantity = 500.00 kg/ha.

Conversion = 1.50E+07 J/kg.

Energy (J) = 500.00 kg/ha ×1.50E+07 J/kg = 7.50E+09 J/ha.

**F3:**

1. Solar energy:

Land area = 10000 m^2^.

Isolation = 3.94E+09 J/m^2^/yr.

Albedo = 20%.

Energy (J) = 3.94E+09 J/m^2^/yr × (1-20%) ×10000 m^2^ = 3.15E+13 J/ha.

1. Rain chemical energy:

Land area = 10000 m^2^.

Rainfall = 1.478 m/yr.

Conversion = 1000 kg/m^3^.

Gibbs free energy = 4940 J/kg.

Energy (J) = 1.478 m/yr × 10000 m^2^ ×1000 kg/m^3^ ×4940 J/kg = 7.31E+10 J/ha.

1. Wind energy:

Land area = 10000 m^2^.

Wind speed = 2.03 m/s.

Air density = 1.3 kg/m^3^.

Drag coefficient = 0.001.

Time (s) = 3.15E+07 s/yr.

Energy (J) = 10000 m^2^ ×1.3 kg/m^3^ × 0.001 × (2.03)^3^ m/s ×3.15E+7 s = 3.43E+09 J/ha.

1. Net soil loss:

Average soil loss = 18.38 t/ha. (Liu et al. 2023)

Organic matter = 1.23 %.

Organic matter energy = 5400 kcal/kg.

Conversion = 4186 J/kcal.

Energy of net soil loss = 1.838E+04 kg/ha × 0.0123 kg/kg soil × 5400 kcal/kg × 4186 J/kcal = 5.11E+09 J/ha.

1. Nitrogen fertilizer:

N content = 3.50E+05 g/ha.

1. Phosphate fertilizer:

P_2_O_5_ content =1.80E+05 g/ha.

1. Potash fertilizer:

K_2_O content = 1.80E+05 g/ha.

1. Pesticides:

Quantity = 2.80E+04 g/ha.

1. Mechanical equipment:

Total use = 2.85E+04 g/yr.

1. Diesel:

Total use = 30.0 kg/ha.

Energy content per kg = 5.53E+07 J/kg. (Jiang et al.2007)

Energy (J) = 30.0 kg/ha × 5.53E+07 J/kg = 1.66E+09 J/ha.

1. Labor:

Hours =3.24E+03 h/ha/yr

1. Seeds:

Corn：

Quantity = 120.00 kg/ha.

Conversion = 1.40E+07 J/kg.

Energy (J) =120.00 kg/ha × 1.40E+07 J/kg =1.68E+09 J/ha.

broad bean：

Quantity = 500.00 kg/ha.

Conversion = 1.50E+07 J/kg.

Energy (J) = 500.00 kg/ha ×1.50E+07 J/kg = 7.50E+09 J/ha.

**F4:**

1. Solar energy:

Land area = 10000 m^2^.

Isolation = 3.94E+09 J/m^2^/yr.

Albedo = 20%.

Energy (J) = 3.94E+09 J/m^2^/yr × (1-20%) ×10000 m^2^ = 3.15E+13 J/ha.

1. Rain chemical energy:

Land area = 10000 m^2^.

Rainfall = 1.478 m/yr.

Conversion = 1000 kg/m^3^.

Gibbs free energy = 4940 J/kg.

Energy (J) = 1.478 m/yr × 10000 m^2^ ×1000 kg/m^3^ ×4940 J/kg = 7.31E+10 J/ha.

1. Wind energy:

Land area = 10000 m^2^.

Wind speed = 2.03 m/s.

Air density = 1.3 kg/m^3^.

Drag coefficient = 0.001.

Time (s) = 3.15E+07 s/yr.

Energy (J) = 10000 m^2^ ×1.3 kg/m^3^ × 0.001 × (2.03)^3^ m/s ×3.15E+7 s = 3.43E+09 J/ha.

1. Net soil loss:

Average soil loss = 18.38 t/ha. (Liu et al. 2023)

Organic matter = 1.23 %.

Organic matter energy = 5400 kcal/kg.

Conversion = 4186 J/kcal.

Energy of net soil loss = 1.838E+04 kg/ha × 0.0123 kg/kg soil × 5400 kcal/kg × 4186 J/kcal = 5.11E+09 J/ha.

1. Nitrogen fertilizer:

N content = 2.24E+05 g/ha.

1. Phosphate fertilizer:

P_2_O_5_ content =1.20E+05 g/ha.

1. Potash fertilizer:

K_2_O content = 1.20E+05 g/ha.

1. Pesticides:

Quantity = 1.70E+04 g/ha.

1. Mechanical equipment:

Total use = 2.85E+04 g/yr.

1. Diesel:

Total use = 30.0 kg/ha.

Energy content per kg = 5.53E+07 J/kg. (Jiang et al.2007)

Energy (J) = 30.0 kg/ha × 5.53E+07 J/kg = 1.66E+09 J/ha.

1. Labor:

Hours =1.80E+03 h/ha/yr

1. Seeds:

Wheat：

Quantity = 600.00 kg/ha.

Conversion = 1.33E+07 J/kg.

Energy (J) = 600.00 kg/ha × 1.33E+07 J/kg = 7.98E+09 J/ha.

Corn：

Quantity = 60.00 kg/ha.

Conversion = 1.40E+07 J/kg.

Energy (J) =60.00 kg/ha × 1.40E+07 J/kg = 8.40E+08 J/ha.

Soybean：

Quantity = 80.00 kg/ha.

Conversion = 1.50E+07 J/kg.

Energy (J) = 80.00 kg/ha ×1.50E+07 J/kg = 1.20E+09 J/ha.

**F5:**

1. Solar energy:

Land area = 10000 m^2^.

Isolation = 3.94E+09 J/m^2^/yr.

Albedo = 20%.

Energy (J) = 3.94E+09 J/m^2^/yr × (1-20%) ×10000 m^2^ = 3.15E+13 J/ha.

1. Rain chemical energy:

Land area = 10000 m^2^.

Rainfall = 1.478 m/yr.

Conversion = 1000 kg/m^3^.

Gibbs free energy = 4940 J/kg.

Energy (J) = 1.478 m/yr × 10000 m^2^ ×1000 kg/m^3^ ×4940 J/kg = 7.31E+10 J/ha.

1. Wind energy:

Land area = 10000 m^2^.

Wind speed = 2.03 m/s.

Air density = 1.3 kg/m^3^.

Drag coefficient = 0.001.

Time (s) = 3.15E+07 s/yr.

Energy (J) = 10000 m^2^ ×1.3 kg/m^3^ × 0.001 × (2.03)^3^ m/s ×3.15E+7 s = 3.43E+09 J/ha.

1. Net soil loss:

Average soil loss = 18.38 t/ha. (Liu et al. 2023)

Organic matter = 1.23 %.

Organic matter energy = 5400 kcal/kg.

Conversion = 4186 J/kcal.

Energy of net soil loss = 1.838E+04 kg/ha × 0.0123 kg/kg soil × 5400 kcal/kg × 4186 J/kcal = 5.11E+09 J/ha.

1. Nitrogen fertilizer:

N content =3.84E+05 g/ha.

1. Phosphate fertilizer:

P_2_O_5_ content = 2.10E+05 g/ha.

1. Potash fertilizer:

K_2_O content = 2.10E+05 g/ha.

1. Pesticides:

Quantity = 2.50E+04 g/ha.

1. Mechanical equipment:

Total use = 2.85E+04 g/yr.

1. Diesel:

Total use = 30.0 kg/ha.

Energy content per kg = 5.53E+07 J/kg. (Jiang et al.2007)

Energy (J) = 30.0 kg/ha × 5.53E+07 J/kg = 1.66E+09 J/ha.

1. Labor:

Hours =2.64E+03 h/ha/yr

1. Seeds:

Wheat：

Quantity = 600.00 kg/ha.

Conversion = 1.33E+07 J/kg.

Energy (J) = 600.00 kg/ha × 1.33E+07 J/kg = 7.98E+09 J/ha.

Corn：

Quantity = 120.00 kg/ha.

Conversion = 1.40E+07 J/kg.

Energy (J) =120.00 kg/ha × 1.40E+07 J/kg = 1.68E+09 J/ha.

References：

Chen, Y., Liu, C., Chen, J., Hu, N., and Zhu, L. (2021b). Assessments on environmental consequences and sustainability of three rice-based rotation systems in Quanjiao, China by an integrated analysis of life cycle, emergy and economic assessment. *J. Clean. Prod*. 310, 127493. doi: 10.1016/j.jclepro.2021.127493

Houshyar, E., Wu, X. F., and Chen, G. Q. (2018). Sustainability of wheat and maize production in the warm climate of southwestern Iran: An emergy analysis. *J. Clean. Prod*. 172, 2246-2255. doi: 10.1016/j.jclepro.2017.11.187

Jiang, M.M., Chen, B., Zhou, J.B., et al., 2007. Emergy account for biomass resource exploitation by agriculture in China. *Ener. Policy* 35(9), 4704-4719. DOI: 10.1016/j.enpol.2007.03.014

Li, T., Chen, W., Liu, F., Yao, H., Huo, Q., Zhang, W., et al. (2023a). Benefits through innovative cropping patterns in the hilly regions of Southwest China: An integrated assessment of emergy and economic returns. *Agronomy*. 13(10), 2640. doi: 10.3390/agronomy13102640

Li, Y., Cai, G., Tan, K., Zeng, R., Chen, X., and Wang, X. (2023b). Emergy- based efficiency and sustainability assessments of diversified multi- cropping systems in South China. *J. Clean. Prod*. 414, 137660. doi: 10.1016/j.jclepro.2023.137660

Liu X, Bao Y, Wang Y, et al., (2023). Spatiotemporal variation characteristics of sediment nutrient load from the soil erosion of the Yangtze River Basin of China from 1901 to 2010. *Ecol. Indic*. 150, 110206. 10.1016/j.ecolind.2023. 110206

Moonilall, N.I., Homenauth, O., and Lal, R. (2020). Emergy analysis for maize fields under different amendment applications in Guyana. *J. Clean. Prod*. 258, 120761. doi: 10.1016/j.jclepro. 2020.120761

Wang, X., Li, Z., Long, P., Yan, L., Gao, W., Chen, Y., et al. (2017). Sustainability assessments of recycling in agricultural systems by emergy accounting. *Resour. Conserv. Recy.* 117, 114-124. doi: 10.1016/j. resconrec. 2016.11.009

Xu, Q., Wang, X., Xiao, B., and Hu, K. (2019). Rice-crab coculture to sustain cleaner food production in Liaohe River Basin, China: An economic and environmental assessment. *J. Clean. Prod.* 208, 188-198. doi: 10.1016/j.jclepro.2018.10.092
